# Population-aware permutation-based significance thresholds for genome-wide association studies

**DOI:** 10.1093/bioadv/vbae168

**Published:** 2024-10-28

**Authors:** Maura John, Arthur Korte, Marco Todesco, Dominik G Grimm

**Affiliations:** Technical University of Munich, TUM Campus Straubing for Biotechnology and Sustainability, Bioinformatics, 94315 Straubing, Germany; Weihenstephan-Triesdorf University of Applied Sciences, Bioinformatics, 94315 Straubing, Germany; Faculty of Biology, University of Würzburg, 97074 Würzburg, Germany; Michael Smith Laboratories, University of British Columbia, Vancouver, BC V6T 1Z4, Canada; Department of Botany and Biodiversity Research Centre, University of British Columbia, Vancouver, BC V6T 1Z4, Canada; Department of Biology, University of British Columbia, Kelowna, BC V1V 1V7, Canada; Technical University of Munich, TUM Campus Straubing for Biotechnology and Sustainability, Bioinformatics, 94315 Straubing, Germany; Weihenstephan-Triesdorf University of Applied Sciences, Bioinformatics, 94315 Straubing, Germany; Technical University of Munich, TUM School of Computation, Information and Technology, 85748 Garching, Germany

## Abstract

**Motivation:**

Permutation-based significance thresholds have been shown to be a robust alternative to classical Bonferroni significance thresholds in genome-wide association studies (GWAS) for skewed phenotype distributions. The recently published method permGWAS introduced a batch-wise approach to efficiently compute permutation-based GWAS. However, running multiple univariate tests in parallel leads to many repetitive computations and increased computational resources. More importantly, traditional permutation methods that permute only the phenotype break the underlying population structure.

**Results:**

We propose permGWAS2, an improved method that does not break the population structure during permutations and uses an elegant block matrix decomposition to optimize computations, thereby reducing redundancies. We show on synthetic data that this improved approach yields a lower false discovery rate for skewed phenotype distributions compared to the previous version and the commonly used Bonferroni correction. In addition, we re-analyze a dataset covering phenotypic variation in 86 traits in a population of 615 wild sunflowers (*Helianthus annuus* L.). This led to the identification of dozens of novel associations with putatively adaptive traits, and removed several likely false-positive associations with limited biological support.

**Availability and implementation:**

permGWAS2 is open-source and publicly available on GitHub for download: https://github.com/grimmlab/permGWAS.

## 1 Introduction

Linear mixed models (LMMs) are a popular method for conducting genome-wide association studies (GWAS) while correcting for confounding factors, such as population structure and cryptic relatedness (Lippert *et al.* 2011, Loh *et al.* 2015, Gumpinger *et al.* 2018). In a typical GWAS, hundreds of thousands to millions of univariate statistical tests must be performed, resulting in a large multiple hypothesis testing problem. Addressing the multiple testing challenge in GWAS often involves managing the family-wise error rate (FWER), which represents the probability of encountering at least one false positive or type I error. A widely used method to approximate the FWER is the Bonferroni correction (Bonferroni 1936). Despite its simplicity, this approach is often criticized for being overly conservative, with an increased risk of false negatives, especially when dealing with normally distributed phenotypes (Eichstaedt *et al.* 2013, Llinares-López *et al.* 2015). The underlying problem stems from the incomplete independence of the genetic markers tested. In general, GWAS are often challenged by the frequent violation of model assumptions, including independence, Gaussian distribution of residuals, and homoscedasticity, as these assumptions are rarely met in real biological data. To overcome some of these limitations, permutation-based methods for GWAS have been introduced to provide a robust alternative to the Bonferroni correction. By empirically estimating the underlying null distribution, one can limit false positive associations in statistical hypothesis testing (Che *et al.* 2014, Nicod *et al.* 2016, John *et al.* 2022). One of the main challenges in using permutation-based approaches is the high computational cost, which was recently addressed in John *et al.* (2022). There we introduced permGWAS, a framework capable of computing efficient batch-wise LMMs using 3- and 4-dimensional tensors. This reformulation, combined with the *maxT* permutation method of Westfall and Young (1993), allowed us to compute permutation-based thresholds when permuting the phenotype in a reasonable amount of time. However, the original version of permGWAS contains only a simple permutation strategy that does not take into account the underlying population structure of the given phenotype. In fact, the original version of permGWAS only permutes the phenotype which breaks not only the correlation between the phenotypic and genotypic values but also the relatedness between the individuals. Furthermore, permGWAS still performs each hypothesis test independently, repeating certain computations that could actually be saved and reused. In fact, when estimating the parameters of an LMM, certain calculations are independent of the marker of interest and can therefore be reused for each single nucleotide polymorphism (SNP).

In this work, we propose permGWAS2, an improved version of permGWAS that uses a new permutation approach that takes into account the population structure during permutations. Furthermore it takes advantage of an elegant block matrix decomposition to reduce the number of redundant computations. We show that our new permutation strategy, which permutes each individual SNP, is as efficient as simply permuting only the phenotype. We also demonstrate in various simulations that permGWAS2 provides a lower false discovery rate (FDR) compared to the previous version and the commonly used Bonferroni correction, especially for phenotypes with highly skewed distributions. Finally, we re-analyze a real dataset covering phenotypic variation in 86 traits in a population of 615 wild sunflowers (*Helianthus annuus* L.) representing the entire natural range of the species. This led to the identification of dozens of novel associations with putatively adaptive traits, and removed several likely false-positive associations with limited biological support.

## 2 Methods

In the following, we first review the basic framework of LMMs and how to estimate effect sizes and variance components, followed by two lemmas on how to efficiently compute permutation-based significance thresholds while taking into account the population structure during permutations.

### 2.1 Linear mixed models

For a vector of n phenotypic observations $y\in R^{n}$, and a matrix $X\in R^{n\times c}$ of c fixed effects including a column of ones for the overall mean, the covariates and the SNP of interest, we consider an LMM of the form

(1) y=Xβ+u+ϵ,

where $\beta \in R^{c}$ denotes the effect sizes of the fixed effects. Here, $u\in R^{n}$ are random effects assumed to follow a zero mean Gaussian distribution with covariance matrix $\sigma_{g}^{2}K$, for genetic variance component $\sigma_{g}^{2}\in R$ and genetic similarity matrix $K\in R^{n\times n}$, and $\epsilon \in R^{n}$ denotes the residual effects, where $\epsilon ∼N\left(0,\sigma_{e}^{2}I\right)$ with residual variance component $\sigma_{e}^{2}\in R$ and identity matrix $I\in R^{n\times n}$. Then y also follows a Gaussian distribution with mean Xβ and covariance matrix $V:=\sigma_{g}^{2}K+\sigma_{e}^{2}I$.Similarly to Lippert *et al.* (2011), we estimate the effect sizes β, and the variance components $\sigma_{g}^{2}$ and $\sigma_{e}^{2}$ by maximizing the log-likelihood function

(2) $$\ell \ell \left(\beta ,\sigma_{g}^{2},\sigma_{e}^{2}\right)=\text{log}\;N\left(y|X\beta ,\sigma_{g}^{2}K+\sigma_{e}^{2}I\right).$$

To speed up the computations, we estimate the variance components only once for a null model without any genetic markers and reuse the estimates for the alternative models including the markers of interest as described in Kang *et al.* (2010). Once we have estimated the variance components, we use an F-test to test the null hypothesis that the marker of interest has no effect on a given phenotype. If the resulting *P*-value is below a predefined significance threshold, we reject the null hypothesis.

Since we test thousands to millions of markers at once in a typical GWAS, we need to correct for these multiple hypotheses to avoid thousands of false-positive associations. One way to do this is to empirically estimate the FWER, i.e. the probability of making at least one false positive, by computing a permutation-based significance threshold (John *et al.* 2022).

### 2.2 Permutation-based significance thresholds

Let m be the number of genetic markers to be tested. To compute a permutation-based significance threshold for a given significance level α∈[0,1], we use the *maxT* method proposed by Westfall and Young (1993). As described in John *et al.* (2022), we first permute the phenotype yq-times and compute the test statistics ${}^{k}t_{i}$ for each permutation k∈{1,…,q} and marker i∈{1,…,m}. Then for each permutation, we take the maximal test statistic over all markers ${}^{k}t_{\text{max}}:=\max_{i\in \{1,\ldots ,m\}}{}^{k}t_{i}$.

For a given marker $s_{i}$ with test statistic $t_{i}$, each maximal test statistic ${}^{k}t_{\text{max}}$ has probability $p_{i}$ of being larger than $t_{i}$. Thus, the number of successes $\sum_{k=1}^{q}1\left({}^{k}t_{\text{max}}\geq t_{i}\right)$ follows a binomial distribution $\text{Bin}(q,p_{i})$, where 1 denotes the indicator function. We can estimate an adjusted permutation-based *P*-value via

(3) $${\hat{p}}_{i}=\frac{\sum_{k=1}^{q}1\left({}^{k}t_{\text{max}}\geq t_{i}\right)}{q}.$$

Then ${\hat{p}}_{i}\leq \alpha$ is equivalent to $t_{i}$ being larger than the (1−α)·100th percentile of the maximal test statistics ${}^{k}t_{\text{max}}$. Thus, the adjusted threshold is given as the α·100th percentile of the corresponding minimal *P*-values ${}^{k}p_{\text{min}}$. This permutation-based threshold is able to control the FWER, as shown in John *et al.* (2022).

#### 2.2.1 Population-aware permutations

The permutation strategy presented above does not take into account the underlying population structure of the given phenotype. In fact, permuting the phenotype breaks not only the correlation between the phenotypic and genotypic values but also the relatedness between the individuals. To preserve this relatedness, one can alternatively compute the covariance matrix anew for each permutation or, equivalently, permute the rows and columns of the covariance matrix using the same permutation as for the phenotype vector. By Lemma 1, this is equivalent to permuting the fixed effects matrix containing the covariates and the SNP of interest. We refer to this permutation method as population-aware permutations. The proof of Lemma 1 can be found in the Supplementary Notes.

Lemma 1 *Consider the LMM fromEquation (1), i.e.* y=Xβ+u+ϵ*with covariance matrix* $V=\sigma_{g}^{2}K+\sigma_{e}^{2}I$*. Let* τ:{1,…,n}→{1,…,n}*be a permutation. Then permuting the entries of the phenotype vector* y*and the rows and columns of* V*with respect to* τ*is equivalent to permuting the rows of* X*with respect to the inverse permutation* $\tau^{-1}$.

#### 2.2.2 Number of permutations

Regardless of the permutation strategy used, we need to choose the number of permutations q to obtain an appropriate permutation-based threshold. For this we use an approach, similar to Che *et al.* (2014). We can write the standard error of the adjusted *P*-value estimate from Equation (3) as $\text{SE}({\hat{p}}_{i})=\sqrt{\frac{\alpha (1-\alpha )}{q}}$. Let γ∈[0,1]. Then $\left[{\hat{p}}_{i}-z_{\gamma }\text{SE}({\hat{p}}_{i}),{\hat{p}}_{i}+z_{\gamma }\text{SE}({\hat{p}}_{i})\right]$ is a γ·100% confidence interval for $p_{i}$, where $z_{\gamma }$ denotes the $(\frac{1+\gamma }{2})\cdot 100$th percentile of the standard normal distribution.

Now let θ∈R be our desired precision in *P*-value estimation. From $z_{\gamma }\text{SE}({\hat{p}}_{i})\leq \theta$, it follows that

(4) $$q\geq z_{\gamma }^{2}\frac{\alpha (1-\alpha )}{\theta^{2}}.$$

Supplementary Table S1 shows the recommended number of permutations for α=0.05 based on Equation (4) for commonly used confidence levels and different precision values.

### 2.3 permGWAS2 architecture

Permutation-based approaches for univariate statistical tests are generally computationally expensive. To accelerate them, we previously introduced batch-wise LMMs (John *et al.* 2022). However, the more complex population-aware permutation strategy requires additional steps to generate batches of permuted SNPs. This comes with even higher costs than the conventional permutation method where only the phenotype gets randomized. Therefore, we propose a block matrix decomposition that reduces the number of repetitive computations and leads to a more efficient implementation.

In the following, we first summarize how to efficiently evaluate the log-likelihood function and review the mathematical framework for batch-wise LMMs based on John *et al.* (2022). Afterward, we show how a block matrix decomposition can be used to compute LMMs more efficiently.

#### 2.3.1 Efficient evaluation of the log-likelihood function

As shown in Lippert *et al.* (2011), the log-likelihood function (2) can be efficiently evaluated using the spectral decomposition of the symmetric kinship matrix $K=\mathrm{UD}U^{⊤}$, where U is an orthogonal matrix of eigenvectors of K and D is a diagonal matrix containing the corresponding eigenvalues. Denote by $\delta :=\frac{\sigma_{e}^{2}}{\sigma_{g}^{2}}$ the ratio of variance components. To simplify the notation we define H:=K+δI and E:=D+δI. Then H can be decomposed as $H=U\left(D+\delta I\right)U^{⊤}=\mathrm{UE}U^{⊤}$. In addition, we introduce the following notation: for each matrix $A\in R^{n\times d}$ with d∈N let $\tilde{A}:=U^{⊤}A$. Then the generalized least squares estimate of the effect sizes can be written as:

(5) $$\begin{array}{cc} \hat{\beta } & ={\left(X^{⊤}H^{-1}X\right)}^{-1}X^{⊤}H^{-1}y\\ ={\left({\tilde{X}}^{⊤}E^{-1}\tilde{X}\right)}^{-1}{\tilde{X}}^{⊤}E^{-1}\tilde{y}. & \end{array}$$

Using $\hat{\beta }$ and the estimate of the genetic variance component ${\hat{\sigma }}_{g}^{2}$, the log-likelihood function (2) can be rewritten as a function only dependent on δ∈R. In order to find the globally optimal δ∈R that maximizes the log-likelihood function we apply Brent’s method to a grid of 100 equidistant values between $10^{-5}$ and $10^{5}$ on a logarithmic scale similarly to Kang *et al.* (2008) and Lippert *et al.* (2011). Finally, we can compute the residual sum of squares via

(6) $$\text{RSS}={\left(\tilde{y}-\tilde{X}\hat{\beta }\right)}^{⊤}E^{-1}\left(\tilde{y}-\tilde{X}\hat{\beta }\right)$$

and calculate the *P*-value.

#### 2.3.2 Batch-wise linear mixed models

Let b be the number of genetic markers to test in parallel. Let $Z\in R^{n\times (c-1)}$ be the matrix containing a column of ones for the intercept and all covariates, and let $s_{i}\in R^{n}$ be the i-th SNP for i∈{1,…,b}. Denote by $X_{i}:=\left[Z,s_{i}\right]\in R^{n\times c}$ the matrix of fixed effects consisting of Z and the SNP $s_{i}$, and let ${\tilde{X}}_{\left(1:b\right)}$ be the 3-dimensional tensor in $R^{b\times n\times c}$ containing the matrices ${\tilde{X}}_{1}$ to ${\tilde{X}}_{b}$ (see Supplementary Fig. S1A). Additionally, for each matrix $A\in R^{n\times d}$ denote by $A_{\left(b\right)}$ the 3-dimensional tensor in $R^{b\times n\times d}$ obtained by stacking b copies of A. Then the effect sizes ${\hat{\beta }}_{\left(1:b\right)}$ and the residual sums of squares ${\text{RSS}}_{\left(1:b\right)}$ for all b SNPs can be computed simultaneously by replacing $\tilde{X}$, E, $\tilde{y}$ and $\hat{\beta }$ in Equations (5) and (6) with ${\tilde{X}}_{\left(1:b\right)}$, $E_{\left(b\right)}$, ${\tilde{y}}_{\left(b\right)}$, and ${\hat{\beta }}_{\left(1:b\right)}$, respectively.

Now let q be the number of permutations. By Lemma 1, population-aware permutations can be obtained by permuting the fixed effects matrices $X_{i}$. Thus, for each permutation $\tau_{k}$ with k∈{1,…,q} and each SNP $s_{i}$ let ${}^{k}X_{i}:=\tau_{k}(X_{i})$ be the fixed effects matrix with permuted rows. Further, denote by ${}^{k}{\tilde{X}}_{\left(1:b\right)}$ again the 3-dimensional tensor containing ${}^{k}{\tilde{X}}_{i}$ for all i. By stacking ${}^{k}{\tilde{X}}_{\left(1:b\right)}$ for all k, we get a 4-dimensional tensor ${}^{\left(1:q\right)}{\tilde{X}}_{\left(1:b\right)}\in R^{q\times b\times n\times c}$. Note that the ratio of variance components ${}^{k}\delta$ and the diagonal matrix ${}^{k}E:=\left(D{+}^{k}\delta I\right)$ need to be recomputed for each permutation $\tau_{k}$. Let ${}^{\left(1:q\right)}E_{\left(b\right)}$ be the 4-dimensional tensor in $R^{q\times b\times n\times n}$ containing the 3-dimensional tensors ${}^{k}E_{\left(b\right)}$ for all k. Further, denote by ${}^{\left(q\right)}{\tilde{y}}_{\left(b\right)}$ the 4-dimensional tensor in $R^{q\times b\times n\times 1}$ obtained by stacking q copies of ${\tilde{y}}_{\left(b\right)}$. Then similarly to before, we can compute the effect sizes ${}^{\left(1:q\right)}{\hat{\beta }}_{\left(1:b\right)}$ and the residual sums of squares ${}^{\left(1:q\right)}{\text{RSS}}_{\left(1:b\right)}$ for b SNPs and q permutations simultaneously by plugging the 4-dimensional tensors ${}^{\left(1:q\right)}{\tilde{X}}_{\left(1:b\right)}$, ${}^{\left(1:q\right)}E_{\left(b\right)}$, ${}^{\left(q\right)}{\tilde{y}}_{\left(b\right)}$ and ${}^{\left(1:q\right)}{\hat{\beta }}_{\left(1:b\right)}$ into Equations (5) and (6).

#### 2.3.3 Efficient block matrix decomposition

To make population-aware permutations more efficient, we introduce a block matrix decomposition in Lemma 2 and show how we can use this to compute ${\hat{\beta }}_{\left(1:b\right)}$ for batch-wise LMMs and ${}^{\left(1:q\right)}{\hat{\beta }}_{\left(1:b\right)}$ for permutations. The proof of Lemma 2 can be found in the Supplementary Notes.

Lemma 2 *Let* $Z\in R^{n\times (c-1)}$*be an arbitrary matrix*, $s\in R^{n}$*a vector, and let* $A\in R^{n\times n}$*be a symmetric matrix. Denote by* $X:=\left[Z,s\right]\in R^{n\times c}$*the matrix obtained by concatenating the vector* s*to the matrix* Z*. Then the matrix* $X^{⊤}\mathrm{AX}\in R^{c\times c}$*has the following block structure*

$$X^{⊤}\mathrm{AX}=\left(\begin{array}{cc} Z^{⊤}\mathrm{AZ} & Z^{⊤}\mathrm{As}\\ {\left(Z^{⊤}\mathrm{As}\right)}^{⊤} & s^{⊤}\mathrm{As} \end{array}\right).$$

Using the notations introduced in the previous paragraphs, note that $X_{i}=U^{⊤}X_{i}=\left[U^{⊤}Z,U^{⊤}s_{i}\right]=\left[\tilde{Z},{\tilde{s}}_{i}\right]$. Similarly to before, denote by ${\tilde{S}}_{\left(1:b\right)}\in R^{b\times n\times 1}$ the 3-dimensional tensor containing the batch of b SNPs ${\tilde{s}}_{1},\ldots ,{\tilde{s}}_{b}$. Note that $E^{-1}$ is a diagonal matrix and thus symmetric. Hence, by transferring Lemma 2 to the 3-dimensional case, we can decompose ${\tilde{X}}_{\left(1:b\right)}^{⊤}E_{\left(b\right)}^{-1}{\tilde{X}}_{\left(1:b\right)}\in R^{b\times c\times c}$ into a block matrix structure containing the 3-dimensional tensors ${\left({\tilde{Z}}^{⊤}E^{-1}\tilde{Z}\right)}_{\left(b\right)}$, ${\left({\tilde{Z}}^{⊤}E^{-1}\right)}_{\left(b\right)}{\tilde{S}}_{\left(1:b\right)}$ and ${\tilde{S}}_{\left(1:b\right)}^{⊤}E_{\left(b\right)}^{-1}{\tilde{S}}_{\left(1:b\right)}$. Since ${\tilde{Z}}^{⊤}E^{-1}\tilde{Z}$ is independent of the SNPs, we only need to compute the parts containing ${\tilde{S}}_{\left(1:b\right)}$ in batches and can assemble the block matrix afterward (see Supplementary Fig. S1B). Similarly, ${\tilde{X}}_{\left(1:b\right)}^{⊤}E_{\left(b\right)}^{-1}{\tilde{y}}_{\left(b\right)}$ can be decomposed into a block structure where only parts are dependent of the SNPs and need to be computed in batches.

Let again q be the number of permutations. Denote by ${}^{k}Z:=\tau_{k}(Z)$ and ${}^{k}s_{i}:=\tau_{k}(s_{i})$ the permuted matrix and vector. Then we have ${}^{k}{\tilde{X}}_{i}=\left[{}^{k}\tilde{Z}{,}^{k}{\tilde{s}}_{i}\right]$. Now when performing GWAS with permutations, we can decompose ${}^{k}{\tilde{X}}_{\left(1:b\right)}^{⊤}{}^{k}E_{\left(b\right)}^{-1}{}^{k}{\tilde{X}}_{\left(1:b\right)}$ as in Lemma 2 for each k. Since ${}^{k}{\tilde{Z}}^{⊤}{}^{k}E^{-1}{}^{k}\tilde{Z}$ only depends on the permutation and not on the SNPs, we only need to compute it once per permutation (see Supplementary Fig. S1C).

Using the described batch-wise LMM with block matrix decomposition, population-aware permutations can be performed as efficiently as conventional permutations with permGWAS, without changing the computed *P*-values. In the following, we refer to the original batch-wise LMM as described in John *et al.* (2022) with conventional permutations as permGWAS and to batch-wise LMMs with block matrix decomposition and population-aware permutations as described above as permGWAS2.

### 2.4 Data

We analyzed the performance of permGWAS2 on publicly available data from *H.annuus*. In addition, we performed experiments on synthetic data to compare population-aware and conventional permutation-based thresholds with the commonly used Bonferroni threshold on differently skewed phenotypes.

#### 2.4.1 Genotypic and phenotypic data

We obtained 86 different phenotypic measurements for 615 *H.annuus* individuals grown in a common garden experiment (Todesco *et al.* 2020) from HeliantHOME, a public database for *H.annuus* phenotypes (Bercovich *et al.* 2022). Fully imputed genotype data with approximately 4.9 million heterozygous SNPs and gene annotation files were downloaded from easyGWAS (Grimm *et al.* 2017) and the Sunflower Genome Database (https://sunflowergenome.org/).

Further, we used 2029 fully imputed homozygous *Arabidopsis thaliana* genotypes (Arouisse *et al.* 2020) with approximately 2.8 million markers for our simulations.

#### 2.4.2 Synthetic phenotypes

We generated several different artificial phenotypes based on *A.thaliana* and *H.annuus* genotypes. First, we sampled 200 individuals from both species and simulated traits using the full data sets as well as filtered data with a minor allele frequency (MAF) >5% resulting in approximately 1.4 and 2.8 million SNPs, respectively. Second, we created synthetic phenotypes for all available 2029 individuals from *A.thaliana*. For each artificial trait, we used a predefined heritability of either 10%, 30%, or 50% and one causal marker explaining either 20% or 2% of the total phenotypic variance. To simulate different phenotypic distributions, we choose either a Gaussian or a Gamma distributed noise with a shape parameter of 0.5, 1, 2, 3, or 4, denoted as N, $\Gamma_{4}$, $\Gamma_{3}$, $\Gamma_{2}$, $\Gamma_{1}$, and $\Gamma_{0.5}$. Note that the smaller the shape parameter, the more skewed the distribution and the longer the tail. Further details of the synthetic data generation can be found in the Supplementary Methods. The exact specifications we used for our simulations are shown in Table 1. For each simulation setting, we generated a total of 100 phenotypes.

**Table 1.** Simulation settings.^a^

Genotype	Samples	HER	MAF	EV	Distribution
*Helianthus annuus*	200	30%	–	20%	N, $\Gamma_{0.5},\ldots ,\Gamma_{4}$
*Helianthus annuus*	200	30%	5%	20%	N, $\Gamma_{0.5},\ldots ,\Gamma_{4}$
*Arabidopsis thaliana*	200	30%	–	20%	N, $\Gamma_{0.5},\ldots ,\Gamma_{4}$
*Arabidopsis thaliana*	200	30%	5%	20%	N, $\Gamma_{0.5},\ldots ,\Gamma_{4}$
*Arabidopsis thaliana*	200	10%	–	20%	N, $\Gamma_{0.5},\ldots ,\Gamma_{4}$
*Arabidopsis thaliana*	200	50%	–	20%	N, $\Gamma_{0.5},\ldots ,\Gamma_{4}$
*Arabidopsis thaliana*	2029	30%	–	2%	N, $\Gamma_{0.5}$

a Specifications for each simulation setting, including the used genotype data, the number of simulated samples, the heritability (HER), the MAF if MAF filtering was used, the explained variance (EV) of the causal SNP, and the distribution of the noise.

### 2.5 Experimental settings

Unless otherwise noted, we ran permGWAS and permGWAS2 for all phenotypes with 500 permutations (based on Supplementary Table S1).

#### 2.5.1 Runtime experiments

To assess the computational performance of permGWAS2 with block matrix decomposition, we compared its runtime with the original permGWAS as well as with the commonly used methods EMMAX (Kang *et al.* 2010), FaST-LMM (Lippert *et al.* 2011), and fastGWA (Jiang *et al.* 2019). We have not compared it to BOLT-LMM (Loh *et al.* 2015) because BOLT-LMM requires genetic coordinates in Morgan units which are not always available, and—according to its manual—should not be used for sample sizes smaller than 5000. We evaluated the runtime with respect to the number of markers, the number of samples, and the number of permutations. To do so, we generated synthetic data with varying numbers of samples and markers by up- and down-sampling a flowering time related phenotype from *A.thaliana* and the corresponding genotype matrix. For each experiment, we took the average of three runs. For EMMAX, FaST-LMM and fastGWA, we used the binary C/C++ implementations. The runtime comparisons were performed on a machine running Ubuntu 22.04.2 LTS with a total of 52 CPUs, 756 GB of memory, and 4 NVIDIA GeForce RTX 3090 GPUs with 24 GB of memory each. For our experiments, we used dedicated Docker containers where we limited the number of CPUs to one core and used a single GPU.

To evaluate the effect of increasing the number of markers, we first fixed the number of samples at 1000 and varied the number of SNPs between $10^{4}$ and $5\times 10^{6}$. In the second experiment, we fixed the number of markers at $10^{6}$ and varied the number of individuals between 100 and $10^{4}$ to analyze how the runtime depends on the number of samples. Finally, to analyze the runtime for permutations, we used 1000 samples and $10^{6}$ markers, and ran between 10 and 500 permutations. Since EMMAX, FaST-LMM, and fastGWA do not support permutations, we took the runtime from the previous experiments and approximated the permutation-based time by multiplying it by the number of permutations. However, this is only an estimate of the runtime without considering the time required for additional pre- and post-processing steps to prepare the data for conducting permutation-based GWAS with these methods.

#### 2.5.2 Assessment of simulation experiments

To analyze the results of our simulation experiments, we defined a phenotype as a true positive (TP) if any marker within a certain window around the causal SNP was considered significant. If a marker outside this window was significant, we classified the phenotype as a false positive (FP). Thus, a phenotype could be both a TP and an FP at the same time. We then calculated the phenotype-wise FDR $\text{pFDR}:=\frac{\text{FP}}{\text{TP}+\text{FP}}$ for each of the six simulation settings using both permutation-based thresholds, Bonferroni and Benjamini-Hochberg. Since linkage disequilibrium (LD) decays on average within 10 kbp in *A.thaliana* (Kim *et al.* 2007) and within 200 bp in *H.annuus* (Liu and Burke 2006), we selected window sizes of 10 kbp and 200 bp for the simulations from *A.thaliana* and *H.annuus*, respectively.

## 3 Results and discussion

### 3.1 Runtime comparisons

**Figure 1.**
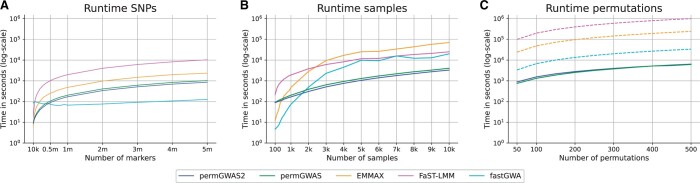
Performance analysis of permGWAS2. Runtime comparisons of permGWAS2 versus permGWAS, EMMAX, FaST-LMM, and fastGWA. Note that all axes are log-scaled. (A) Computational time as function of number of SNPs with fixed number of 1000 samples. (B) Computational time as function of number of samples with $10^{6}$ marker each. (C) Computational time as function of number of permutations with 1000 samples and $10^{6}$ marker each. Dashed lines for EMMAX, FaST-LMM, and fastGWA are estimated based on the computational time for 1000 samples and $10^{6}$ markers times the number of permutations. permGWAS and permGWAS2 were run on a single GPU.

Our runtime comparisons in Fig. 1A–C show that the performance of permGWAS2 is similar to that of permGWAS for all three experiments. For all models except fastGWA, the runtime increases linearly with respect to the number of markers (see Fig. 1A). Here both permGWAS versions clearly outperform EMMAX and FaST-LMM with a runtime of around 14 min for permGWAS2 for 5 million SNPs. However, with a runtime of approximately 2 min, fastGWA shows superior performance. For large sample sizes the computational runtime of permGWAS2 and permGWAS show also a nearly linear dependency both being an order of magnitude faster than the competitors with around 55 min for 10k samples with permGWAS2 (see Fig. 1B). When using permutations, both permGWAS versions significantly outperform all of the competitors (see Fig. 1C), with about 100 min for both permGWAS versions, more than 9 h for fastGWA and several days for EMMAX and FaST-LMM for 500 permutations. This is remarkable considering that permGWAS2 permutes the fixed effects matrix for all genetic markers instead of only permuting the phenotype vector as in the first permGWAS version.

### 3.2 Results in simulated data

**Figure 2.**
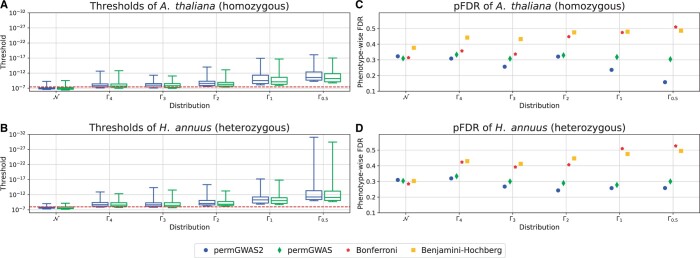
Comparison of permutation-based thresholds with Bonferroni and Benjamini–Hochberg on simulated data with different distributions (N, $\Gamma_{4}$, $\Gamma_{3}$, $\Gamma_{2}$, $\Gamma_{1}$, $\Gamma_{0.5}$) for a heritability of 30% without MAF filtering. (A and B) Permutation-based thresholds over 100 simulations as box plots. Static Bonferroni threshold as red dashed line. (A) Thresholds for homozygous *Arabidopsis thaliana* data. (B) Thresholds for heterozygous *Helianthus annuus* data. (C and D) Phenotype-wise FDR for both permutation-based thresholds, Bonferroni and Benjamini–Hochberg over 100 simulations. (C) pFDR for homozygous *A.thaliana* data. (D) pFDR for heterozygous *H.annuus* data.

Figure 2A and B show the permutation-based significance thresholds for the simulations with 200 samples, 30% heritability and without MAF filtering as boxplots compared to the static Bonferroni threshold. The results with MAF greater than 5% are shown in Supplementary Fig. S2A and B. For all settings in both species, the thresholds with permGWAS2, i.e. with the population-aware permutations, tend to be slightly more stringent than the thresholds with the conventional permutation strategy of permGWAS. In general, both permutation-based thresholds are less conservative than Bonferroni for normally distributed phenotypes, and become more stringent the more skewed the distribution is. For Gamma distributions with larger shape parameters, i.e. for $\Gamma_{4}$ and $\Gamma_{3}$, the thresholds for *A.thaliana* phenotypes seem to fluctuate around the Bonferroni thresholds, while they are much stricter for *H.annuus*. Overall, the thresholds in *H.annuus* tend to be stricter than those in *A.thaliana*. However, this could also be due to the different number of markers tested. In particular, when no MAF filtering is used, the thresholds for both species vary to a greater extent compared to a MAF of 5%. In addition, the thresholds tend to be stricter without filtering, which may also be due to the increased number of SNPs tested.

Figure 2C and D summarize the pFDRs for permGWAS2, permGWAS, Bonferroni, and the FDR-based Benjamini–Hochberg method for the simulations without MAF filtering. For the homozygous data, the pFDR with permGWAS2 decreases visibly for smaller shape parameters, while the pFDR with conventional permutations seems to be stable between 0.3 and 0.35. Whereas the pFDR for *H.annuus* decreases slightly for both permutation approaches with smaller values for permGWAS2. In contrast, in both species the Bonferroni and Benjamini–Hochberg pFDRs both increase as the simulations become more skewed, reaching 0.5 for the most extreme cases. Thus, in these cases we get as many false positives as false negatives with both thresholds. In comparison, when using an MAF of 5% for *A.thaliana* (see Supplementary Fig. S2C), both permutation-based methods and Bonferroni show stable performance between 0.2 and 0.3, except for the more skewed phenotypes, where the pFDR increases with Bonferroni. Again, Benjamini–Hochberg shows the largest pFDR values in all cases. The pFDRs with both permutation-based thresholds are quite similar for the heterozygous *H.annuus* data with MAF >5% (see Supplementary Fig. S2D), with slightly smaller values for permGWAS2 and show a clear advantage in controlling the pFDR over commonly used methods such as Bonferroni and Benjamini–Hochberg. These results suggest that MAF filtering improves the performance of Bonferroni, while population-aware permutations are better at dealing with rare variants.

**Figure 3.**
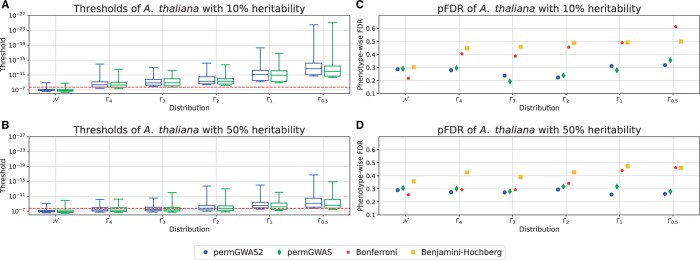
Comparison of permutation-based thresholds with Bonferroni and Benjamini–Hochberg on simulated data with different distributions (N, $\Gamma_{4}$, $\Gamma_{3}$, $\Gamma_{2}$, $\Gamma_{1}$, $\Gamma_{0.5}$) and different heritabilities. (A and B) Permutation-based thresholds over 100 simulations as box plots. Static Bonferroni threshold as red dashed line. (A) Thresholds for *Arabidopsis thaliana* data with 10% heritability. (B) Thresholds for *A.thaliana* data with 50% heritability. (C and D) Phenotype-wise FDR for both permutation-based thresholds, Bonferroni and Benjamini–Hochberg over 100 simulations. (C) pFDR for *A.thaliana* data with 10% heritability. (D) pFDR for *A.thaliana* data with 50% heritability.

Figure 3A–D show the thresholds and pFDRs of the *A.thaliana* simulations with heritabilities of 10% and 50%. Similarly to the simulations with heritability of 30%, the thresholds get more stringent the more skewed the distribution. In addition, the thresholds for the gamma distributed phenotypes in general are even more stringent for smaller heritabilities. This is not surprising, since the influence of the noise on the total phenotypic distribution increases with smaller heritabilities. Indeed, for a heritability of 50%, gamma distributed phenotypes with bigger shape parameters show pFDRs similar to normally distributed phenotypes, where the Bonferroni threshold and the permutation-based thresholds perform en par. In general, the pFDRs with permutation-based thresholds seem to be quite stable, whereas the pFDR with Bonferroni increases faster for smaller heritabilities.

The thresholds and pFDRs for the simulations with 2029 samples are shown in Supplementary Fig. S3A and B. Similarly to the results of *A.thaliana* with 200 samples in Fig. 2A and C, both permutation-based thresholds are less conservative than Bonferroni for the normally distributed phenotypes and more stringent for the skewed phenotypes with shape parameter 0.5. Again, thresholds with permGWAS2 are even more stringent than with permGWAS. The pFDRs also show a similar pattern, where permGWAS2, permGWAS, and Bonferroni perform en par with pFDRs around 0.3, slightly outperforming Benjamini-Hochberg. For the skewed phenotypes, permGWAS2 shows the smallest pFDR of approximately 0.15, whereas the pFDR with Bonferroni and Benjamini-Hochberg are again bigger than 0.5. Note here that the more stringent thresholds with permGWAS2 identify less significant hits in general for the gamma distributed phenotypes than Bonferroni. However, the significant associations they find are more likely to be TPs in contrast to Bonferroni and Benjamini–Hochberg.

### 3.3 Results in *Helianthus annuus*

After emphasizing the benefits of employing a population-aware permutation-based threshold using simulated data, we examine a dataset of 86 phenotypes for wild common sunflower (*H.annuus*). Common sunflower is the closest wild relative to cultivated sunflower (*H.annuus var. macrocarpa*), one of the main global oilcrops (FAO 2022). An obligate outcrosser, wild *H.annuus* is found across most of North America, with populations adapted to a variety of different environments. This is a relatively challenging dataset for GWAS, as *H.annuus* has a large, repetitive genome (3.1 Gbp) (Badouin *et al.* 2017). Wild individuals are highly heterozygous, with rapidly decaying LD. While population structure in wild *H.annuus* is largely shaped by isolation by distance, a very distinctive ecotype is found in Texas (Todesco *et al.* 2020). Accurate characterization of genotype-phenotype associations in this dataset is doubly relevant: it provides insights into the genetic basis of environmental adaptation in this species—and in plants in general—and identifies alleles that can contribute to improvement of yield- and resistance-related traits in cultivated sunflower. We used permGWAS2 and permGWAS to compute adjusted permutation-based significance thresholds with 500 permutations each for all 86 phenotypes. A 5% MAF filter was applied to each experiment.

For eight traits, the implementation of population-aware permutation-based thresholds led to the identification of 222 additional significant associations compared to Bonferroni-corrected thresholds. Many of these novel associations were for traits related to flowering time, such as total leaf number (Supplementary Fig. S4A and B) and days to budding (Supplementary Fig. S4C and D), which have distinctly non-normal distributions with long tails. For example, a significant association with time to budding was found for a SNP within the Ha412HOChr03g0100731 gene, which is predicted to encode a trehalose 6 phosphate synthase (TPS); TPSs have been shown to play a fundamental role in regulating flowering through the so-called autonomous pathway (Wahl *et al.* 2013). Other SNPs associated with variation in the number of branches on the main stem of the plant fell into the sunflower homolog (Ha412HOChr08g0368051) of a gene known to regulate the rate at which new organs are produced in *A.thaliana* (*KLUH*) (Wang *et al.* 2008) and rice (*PLA1*) (Miyoshi *et al.* 2004).

**Figure 4.**
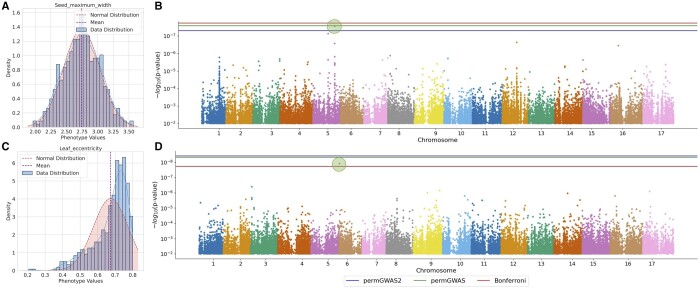
Density and Manhattan plots of two real *Helianthus annuus* phenotypes. (A) Phenotype density plot of the normally distributed phenotype *seed maximum width* (Shapiro–Wilk test *P*-value: 0.844). (B) Corresponding Manhattan plot with one significant hit on chromosome 5. Bonferroni threshold too stringent. (C) Phenotype density plot of the non-normally distributed phenotype *leaf eccentricity* (Shapiro–Wilk test *P*-value: 6.28e−21). (D) Corresponding Manhattan plot with one significant hit on chromosome 6. Bonferroni threshold not stringent enough.

Interestingly, we were also able to identify significant associations for four traits that had none using Bonferroni-based significance thresholds. Two of these also had no significant hits using the simple permutation-based threshold of the original permGWAS. One of these newly identified SNPs is associated with seed size (Fig. 4A and B) and falls within the sequence of a gene (Ha412HOChr05g0230551) predicted to encode a phospholipase; phospholipases are known to be involved in seed lipid metabolism and germination (Scherer *et al.* 2010). Remarkably, for one phenotype—a leaf height measure—we were able to identify a significant association only with the population-unaware permutation threshold. In general, many leaf shape traits have less stringent thresholds for the simpler permutation strategy than for the population-aware permutations.

Finally, our permutation-based approach sometimes produced stricter significance thresholds, resulting in fewer associations for 16 traits. In most cases, this affected highly derived leaf or seed shape traits (e.g. leaf eccentricity) that are unlikely to have direct biological relevance, or very noisy measures of trichome (i.e. leaf hair) density (Fig. 4C and D). In these cases, it seems likely that the stricter thresholds obtained with permGWAS2 will remove spurious associations.

A summary, including the number of significant associations for each trait, and detailed lists of identified hits and corresponding genes for each threshold can be found in the Supplementary Results, including Supplementary Figs S5–S33 and Supplementary Tables S2–S30.

## 4 Conclusions

We introduced permGWAS2, an improved and accelerated GWAS method for computing permutation-based adjusted significance thresholds that do not break the population structure during permutation. We proved that permuting the phenotype and the corresponding covariance matrix is indeed equivalent to permuting the fixed effects matrix including the SNP of interest. Although permuting all markers is computationally more expensive than simply randomizing the phenotype vector, we showed that permGWAS2 is equivalent in runtime to the original permGWAS version due to an elegant block matrix decomposition. Our results show that permGWAS2 provides a lower FDR compared to its predecessor and the traditional Bonferroni correction for skewed phenotypic distributions. In addition, we re-analyze 86 traits in a population of 615 wild sunflowers, leading to the identification of dozens of novel associations with putatively adaptive traits and the removal of several likely false-positive associations with limited biological support.

## Supplementary Material

vbae168_Supplementary_Data

## Data Availability

permGWAS2 is open-source and publicly available on GitHub for download: https://github.com/grimmlab/permGWAS.
