# Supplementary material for: Population-aware permutation-based significance thresholds for genome-wide association studies
Source: Bioinform Adv. 2024 Oct 28;4(1):vbae168. doi: 10.1093/bioadv/vbae168 (PMC11639184; doi:10.1093/bioadv/vbae168)
Supplement: vbae168_Supplementary_Data [file vbae168_supplementary_data.pdf]

# Supplementary Information

## Population-aware permutation-based significance thresholds for genome-wide association studies

Maura John[1,2], Arthur Korte[3], Marco Todesco[4,5,6] and Dominik G. Grimm[1,2,7,*]

[1]Technical University of Munich, Campus Straubing for Biotechnology and Sustainability, Bioinformatics, Straubing, Germany
[2]Weihenstephan-Triesdorf University of Applied Sciences, Bioinformatics, Straubing, Germany
[3]University of Würzburg, Faculty of Biology, Würzburg, Germany
[4]University of British Columbia, Michael Smith Laboratories, Vancouver BC, Canada
[5]University of British Columbia, Department of Botany and Biodiversity Research Centre, Vancouver BC, Canada
[6]University of British Columbia, Department of Biology, Kelowna BC, Canada
[7]Technical University of Munich, TUM School of Computation, Information and Technology, Garching, Germany

* Corresponding authors: dominik.grimm@hswt.de

**Author Contributions**: M.J. and D.G.G. conceived the study and experiment(s), M.J. and D.G.G. conducted the experiment(s), M.J., A.K., M.T. and D.G.G. analysed the results. M.J., M.T. and D.G.G. wrote the manuscript. All authors reviewed the manuscript.

## Contents

**1 Supplementary Notes** **2**
1.1 Proof of Lemma 1 . . . . . 2
1.2 Proof of Lemma 2 . . . . . 3
1.3 Synthetic Data Generation . . . . . 3
1.4 Implementation & Availability . . . . . 4

**2 Supplementary Tables** **5**

**3 Supplementary Figures** **5**

**4 Supplementary Results** **8**

# 1 Supplementary Notes

## 1.1 Proof of Lemma 1

*Proof.* Let $P_\tau \in \mathbb{R}^{n\times n}$ denote the permutation matrix obtained by permuting the rows of the identity matrix $\boldsymbol{I} \in \mathbb{R}^{n\times n}$ according to $\tau$. Note that permutation matrices are orthogonal, i.e., $\boldsymbol{P}_\tau \boldsymbol{P}_\tau^\top = \boldsymbol{I}$, and that $\boldsymbol{P}_\tau^\top = \boldsymbol{P}_\tau^{-1} = \boldsymbol{P}_{\tau^{-1}}$ is the permutation matrix of the inverse permutation $\tau^{-1}$. Then multiplying a matrix $\boldsymbol{A}$ with $\boldsymbol{P}_\tau$ from left permutes the rows of $\boldsymbol{A}$ and multiplying it from right with $\boldsymbol{P}_\tau^\top$ permutes the columns of $\boldsymbol{A}$ according to $\tau$. For the LMM in Equation (1) the log-likelihood function in (2) is given as

$$\ell\ell\left(\boldsymbol{\beta}, \sigma_g^2, \sigma_e^2\right) = -\frac{1}{2}\Big(n\log(2\pi) + \log|\boldsymbol{V}| + (\boldsymbol{y} - \boldsymbol{X\beta})^\top \boldsymbol{V}^{-1}(\boldsymbol{y} - \boldsymbol{X\beta})\Big).$$

Now consider the LMM $\boldsymbol{P}_\tau \boldsymbol{y} = \boldsymbol{X\beta} + \boldsymbol{u} + \boldsymbol{\epsilon}$ with covariance matrix $\boldsymbol{P}_\tau \boldsymbol{V} \boldsymbol{P}_\tau^\top$. Plugging it into the equation above, the log-likelihood function changes to

$$\begin{aligned}
\ell\ell\left(\boldsymbol{\beta}, \sigma_g^2, \sigma_e^2\right) &= \log\mathcal{N}\left(\boldsymbol{P}_\tau \boldsymbol{y} \mid \boldsymbol{X\beta}, \boldsymbol{P}_\tau \boldsymbol{V} \boldsymbol{P}_\tau^\top\right)\\
&= -\frac{1}{2}\Big(n\log(2\pi) + \log\left|\boldsymbol{P}_\tau \boldsymbol{V} \boldsymbol{P}_\tau^\top\right| + (\boldsymbol{P}_\tau\boldsymbol{y} - \boldsymbol{X\beta})^\top \left(\boldsymbol{P}_\tau \boldsymbol{V} \boldsymbol{P}_\tau^\top\right)^{-1}(\boldsymbol{P}_\tau\boldsymbol{y} - \boldsymbol{X\beta})\Big)\\
&= -\frac{1}{2}\Big(n\log(2\pi) + \log|\boldsymbol{V}| + (\boldsymbol{P}_\tau\boldsymbol{y} - \boldsymbol{X\beta})^\top \boldsymbol{P}_\tau \boldsymbol{V}^{-1} \boldsymbol{P}_\tau^\top (\boldsymbol{P}_\tau\boldsymbol{y} - \boldsymbol{X\beta})\Big)\\
&= -\frac{1}{2}\Big(n\log(2\pi) + \log|\boldsymbol{V}| + \left(\boldsymbol{y} - \boldsymbol{P}_\tau^\top\boldsymbol{X\beta}\right)^\top \boldsymbol{V}^{-1}\left(\boldsymbol{y} - \boldsymbol{P}_\tau^\top\boldsymbol{X\beta}\right)\Big)\\
&= \log\mathcal{N}\left(\boldsymbol{y} \mid \boldsymbol{P}_\tau^\top\boldsymbol{X\beta}, \boldsymbol{V}\right),
\end{aligned}$$

which is the log likelihood function of the LMM $y = \boldsymbol{P}_\tau^\top \boldsymbol{X\beta} + \boldsymbol{u} + \boldsymbol{\epsilon}$ with covariance matrix $\boldsymbol{V}$. Similarly, the restricted log likelihood

$$\ell\ell_{REML}\left(\boldsymbol{\beta}, \sigma_g^2, \sigma_e^2\right) = \ell\ell_{ML} + \frac{1}{2}\Big(c\log(2\pi) + \log\left|\boldsymbol{X}^\top\boldsymbol{X}\right| - \log\left|\boldsymbol{X}^\top\boldsymbol{V}^{-1}\boldsymbol{X}\right|\Big),$$

with $\ell\ell_{ML} := \ell\ell\left(\boldsymbol{\beta}, \sigma_g^2, \sigma_e^2\right)$, changes to

$$\begin{aligned}
\ell\ell_{REML}\left(\boldsymbol{\beta}, \sigma_g^2, \sigma_e^2\right) &= \ell\ell_{ML} + \frac{1}{2}\Big(c\log(2\pi) + \log\left|\boldsymbol{X}^\top\boldsymbol{X}\right| - \log\left|\boldsymbol{X}^\top\left(\boldsymbol{P}_\tau\boldsymbol{V}\boldsymbol{P}_\tau^\top\right)^{-1}\boldsymbol{X}\right|\Big)\\
&= \ell\ell_{ML} + \frac{1}{2}\left(c\log(2\pi) + \log\left|\boldsymbol{X}^\top\boldsymbol{P}_\tau\boldsymbol{P}_\tau^\top\boldsymbol{X}\right| - \log\left|\boldsymbol{X}^\top\boldsymbol{P}_\tau\boldsymbol{V}^{-1}\boldsymbol{P}_\tau^\top\boldsymbol{X}\right|\right)\\
&= \ell\ell_{ML} + \frac{1}{2}\Big(c\log(2\pi) + \log\left|\left(\boldsymbol{P}_\tau^\top\boldsymbol{X}\right)^\top\boldsymbol{P}_\tau^\top\boldsymbol{X}\right| - \log\left|\left(\boldsymbol{P}_\tau^\top\boldsymbol{X}\right)^\top\boldsymbol{V}^{-1}\boldsymbol{P}_\tau^\top\boldsymbol{X}\right|\Big).
\end{aligned}$$

□

## 1.2 Proof of Lemma 2

*Proof.* Let $\boldsymbol{Z} = (z_{ij})_{\substack{i=1,\dots,n\\ j=1,\dots,c-1}}$, $\boldsymbol{A} = (a_{it})_{\substack{i=1,\dots,n\\ t=1,\dots,n}}$, $\boldsymbol{s} = (s_i)_{i=1,\dots,n}$ and $\boldsymbol{X} = (x_{ik})_{\substack{i=1,\dots,n\\ k=1,\dots,c}}$. Then

$$x_{ik} = \begin{cases} z_{ij} & \text{if } k = j \in \{1,\dots,c-1\} \\ s_i & \text{if } k = c \end{cases}$$

Denote the transposed matrices by $\boldsymbol{X}^\top = (x'_{ki})_{\substack{k=1,\dots,c\\ i=1,\dots,n}}$ and $\boldsymbol{Z}^\top = \left(z'_{ji}\right)_{\substack{j=1,\dots,c-1\\ i=1,\dots,n}}$, where $x'_{ki} = x_{ik}$ and $z'_{ji} = z_{ij}$. It follows that

$$\boldsymbol{X}^\top \boldsymbol{A} \boldsymbol{X} = \left(x'_{li}\right)_{\substack{l=1,\dots,c\\ i=1,\dots,n}} \cdot (a_{it})_{\substack{i=1,\dots,n\\ t=1,\dots,n}} \cdot (x_{tk})_{\substack{t=1,\dots,n\\ k=1,\dots,c}} = \left(\sum_{t=1}^{n}\left(\sum_{i=1}^{n} x'_{li} a_{it}\right) x_{tk}\right)_{\substack{l=1,\dots,c\\ k=1,\dots,c}} \in \mathbb{R}^{c\times c}$$

with

$$\sum_{t=1}^{n}\left(\sum_{i=1}^{n} x'_{li} a_{it}\right) x_{tk} = \begin{cases} \sum_{t=1}^{n}\left(\sum_{i=1}^{n} z'_{li} a_{it}\right) z_{tk} & \text{if } l,k < c \\ \sum_{t=1}^{n}\left(\sum_{i=1}^{n} z'_{li} a_{it}\right) s_t & \text{if } l < c \text{ and } k = c \\ \sum_{t=1}^{n}\left(\sum_{i=1}^{n} s_i a_{it}\right) z_{tk} & \text{if } k < c \text{ and } l = c \\ \sum_{t=1}^{n}\left(\sum_{i=1}^{n} s_i a_{it}\right) s_t & \text{if } l = k = c \end{cases}$$

Finally, the claim follows from:

$$\boldsymbol{Z}^\top \boldsymbol{A} \boldsymbol{Z} = \left(\sum_{t=1}^{n}\left(\sum_{i=1}^{n} z'_{hi} a_{it}\right) z_{tj}\right)_{\substack{h=1,\dots,c-1\\ j=1,\dots,c-1}} \in \mathbb{R}^{(c-1)\times(c-1)},$$

$$\boldsymbol{s}^\top \boldsymbol{A} \boldsymbol{s} = \sum_{t=1}^{n}\left(\sum_{i=1}^{n} s_i a_{it}\right) s_t \in \mathbb{R},$$

$$\boldsymbol{Z}^\top \boldsymbol{A} \boldsymbol{s} = \left(\sum_{t=1}^{n}\left(\sum_{i=1}^{n} z'_{ji} a_{it}\right) s_t\right)_{j=1,\dots,c-1} \in \mathbb{R}^{(c-1)\times 1},$$

$$\boldsymbol{s}^\top \boldsymbol{A} \boldsymbol{Z} = \left(\boldsymbol{Z}^\top \boldsymbol{A} \boldsymbol{s}\right)^\top \in \mathbb{R}^{1\times(c-1)}.$$

□

## 1.3 Synthetic Data Generation

To create artificial phenotypes with a heritability of $h \in [0,1]$, we sampled $n$ individuals from fully imputed genomic data. For each simulation $\boldsymbol{y} \in \mathbb{R}^n$, we first computed the realized relationship kernel as a kinship matrix and its Cholesky decomposition $\boldsymbol{K} = \boldsymbol{C}\boldsymbol{C}^\top$. To simulate the polygenic background we chose a random vector $\boldsymbol{u} \in \mathbb{R}^n$ where each element was drawn from a Gaussian distribution with zero mean and a variance of 1, and multiplied it by $\boldsymbol{C}$ so that $\mathrm{Var}(\boldsymbol{C}\boldsymbol{u}) = \boldsymbol{K}$. Next, we added a random noise vector $\boldsymbol{\epsilon} \in \mathbb{R}^n$ drawn from either a Gamma or a zero mean normal distribution such

that the noise contributed $(1-h)\cdot 100\%$ to the total phenotypic variance. To analyze the effects of differently skewed phenotypes, we used shape parameters of 0.5, 1, 2, 3, and 4 for the Gamma distribution, where the smaller the shape parameter, the more skewed the phenotype distribution becomes with longer tails. Finally, we added a causal SNP $\boldsymbol{s}$ with an effect size $\beta$ that explained either 20% or 2% of the phenotypic variance. Thus, each artificial phenotype was given as $\boldsymbol{y} = \beta\boldsymbol{s} + \boldsymbol{C}\boldsymbol{u} + \boldsymbol{\epsilon}$. For each setting, we generated 100 simulations.

### 1.4 Implementation & Availability

`permGWAS2` is implemented in Python3 as a standalone command-line tool. It uses `PyTorch` (Paszke *et al.*, 2019) in addition to common scientific computing libraries such as `numpy` (Harris *et al.*, 2020), `pandas` (McKinney *et al.*, 2011), and `scipy` (Virtanen *et al.*, 2020) to support multi-core and GPU usage as well as efficient tensor arithmetic. We support several common genotype and phenotype file formats, including PLINK (Purcell *et al.*, 2007; Chang *et al.*, 2015), CSV, and HDF5. For the genetic similarity matrix, the user can either provide a precomputed matrix or use the implemented realized relationship kernel, which is computed by `permGWAS2` by default. In addition, the user can specify covariates to account for certain fixed effects. To estimate the variance components, `permGWAS2` includes a custom optimization function using Brent's method. Per default, `permGWAS2` computes population-aware permutations. However, it is also possible to choose the permutation strategy of the original `permGWAS` where only the phenoytpe $\boldsymbol{y}$ is permuted. To simplify the workflow, `permGWAS2` supports the use of YAML configuration files. Our framework also includes functions to visualize p-values as Manhattan or QQ-plots. Our code is open source and publicly available on GitHub: https://github.com/grimmlab/permGWAS.

## References

Chang, C. C., Chow, C. C., Tellier, L. C., Vattikuti, S., Purcell, S. M., and Lee, J. J. (2015). Second-generation plink: rising to the challenge of larger and richer datasets. *GigaScience*, **4**(1), 7.

Harris, C. R., Millman, K. J., Van Der Walt, S. J., Gommers, R., Virtanen, P., *et al.* (2020). Array programming with numpy. *Nature*, **585**(7825), 357–362.

McKinney, W. *et al.* (2011). pandas: a foundational python library for data analysis and statistics. *Python for high performance and scientific computing*, **14**(9), 1–9.

Paszke, A., Gross, S., Massa, F., Lerer, A., Bradbury, J., *et al.* (2019). Pytorch: An imperative style, high-performance deep learning library. *Advances in neural information processing systems*, **32**.

Purcell, S., Neale, B., Todd-Brown, K., Thomas, L., Ferreira, M. A., *et al.* (2007). Plink: a tool set for whole-genome association and population-based linkage analyses. *The American journal of human genetics*, **81**(3), 559–575.

Virtanen, P., Gommers, R., Oliphant, T. E., Haberland, M., Reddy, T., *et al.* (2020). Scipy 1.0: fundamental algorithms for scientific computing in python. *Nature methods*, **17**(3), 261–272.

## 2 Supplementary Tables

| $\theta$ \ $\gamma \cdot 100\%$ | 68% | 90% | 95% | 99% |
|---|---|---|---|---|
| 0.005 | 1900 | 5173 | 7300 | 12648 |
| 0.01 | 475 | 1294 | 1825 | 3162 |
| 0.02 | 119 | 324 | 457 | 791 |
| 0.05 | 19 | 52 | 73 | 127 |

**Supplementary Table 1: Number of permutations:** Recommended minimal number of permutations with a significance level of $\alpha = 0.05$ for different precision values $\theta$ and confidence levels $\gamma \cdot 100\%$.

## 3 Supplementary Figures

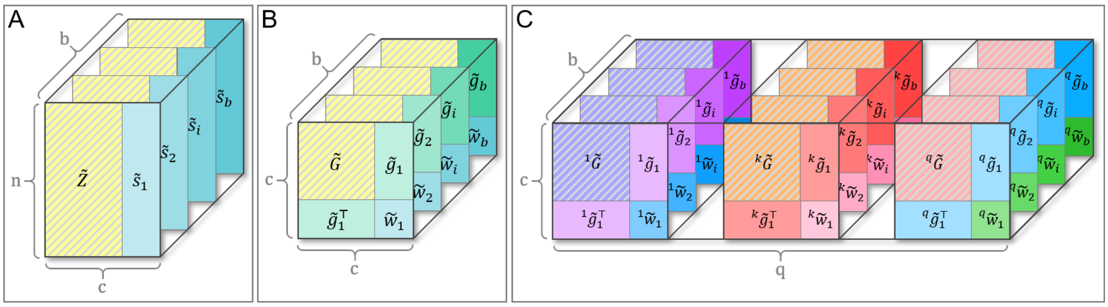


**Supplementary Figure 1: Schematic visualization of tensors and block matrices of the `permGWAS2` architecture.** Note that shaded areas are the same in each layer within a 3D tensor. (A) 3D tensor $\widetilde{X}_{(1:b)} \in \mathbb{R}^{b \times n \times c}$ containing the fixed effects matrices $\widetilde{X}_i = \left[\widetilde{Z}, \widetilde{s}_i\right] \in \mathbb{R}^{n \times c}$ with SNPs $s_i$ for $i \in \{1, \ldots, b\}$. (B) 3D representation of block matrix structure with $\widetilde{G} := \widetilde{Z}^\top E^{-1} \widetilde{Z}$, $\widetilde{g}_i := \widetilde{Z}^\top E^{-1} \widetilde{s}_i$ and $\widetilde{w}_i := \widetilde{s}_i^\top E^{-1} \widetilde{s}_i$ for $i \in \{1, \ldots, b\}$. (C) 4D representation of permutation-based block matrix structure with ${}^k\widetilde{G} := {}^k\widetilde{Z}^\top\, {}^kE^{-1}\, {}^k\widetilde{Z}$, ${}^k\widetilde{g}_i := {}^k\widetilde{Z}^\top\, {}^kE^{-1}\, {}^k\widetilde{s}_i$ and ${}^k\widetilde{w}_i := {}^k\widetilde{s}_i^\top\, {}^kE^{-1}\, {}^k\widetilde{s}_i$ for $i \in \{1, \ldots, b\}$ an $k \in \{1, \ldots, q\}$.

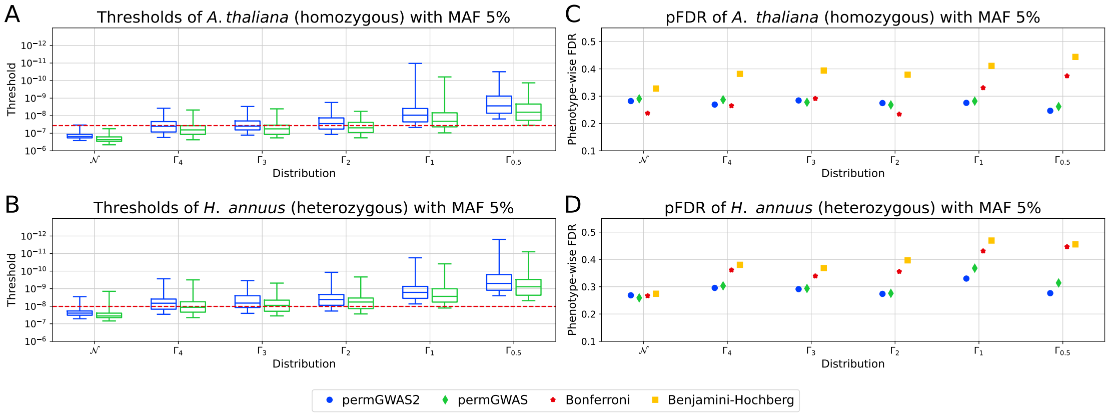


**Supplementary Figure 2: Comparison of permutation-based thresholds with Bonferroni and Benjamini-Hochberg on simulated data with MAF greater 5% and different distributions ($\mathcal{N}$, $\Gamma_4$, $\Gamma_3$, $\Gamma_2$, $\Gamma_1$, $\Gamma_{0.5}$):** (A)-(B) Permutation-based thresholds over 100 simulations as box plots. Static Bonferroni threshold as red dashed line. (A) Thresholds for homozygous *A. thaliana* data. (B) Thresholds for heterozygous *H. annuus* data. (C)-(D) Phenotype-wise FDR for both permutation-based thresholds, Bonferroni and Benjamini-Hochberg over 100 simulations. (C) pFDR for homozygous *A. thaliana* data. (D) pFDR for heterozygous *H. annuus* data.

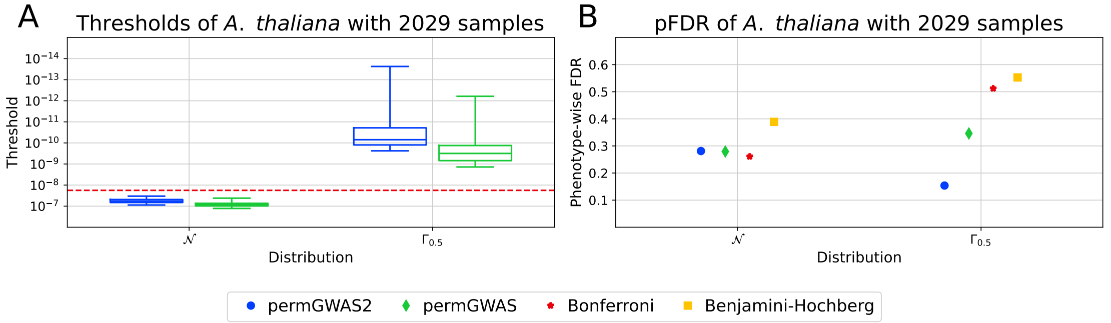


**Supplementary Figure 3: Comparison of permutation-based thresholds with Bonferroni and Benjamini-Hochberg on simulated data with 2029 samples and different distributions ($\mathcal{N}$, $\Gamma_{0.5}$):** (A) Permutation-based thresholds over 100 simulations as box plots. Static Bonferroni threshold as red dashed line. (B) Phenotype-wise FDR for both permutation-based thresholds, Bonferroni and Benjamini-Hochberg over 100 simulations.

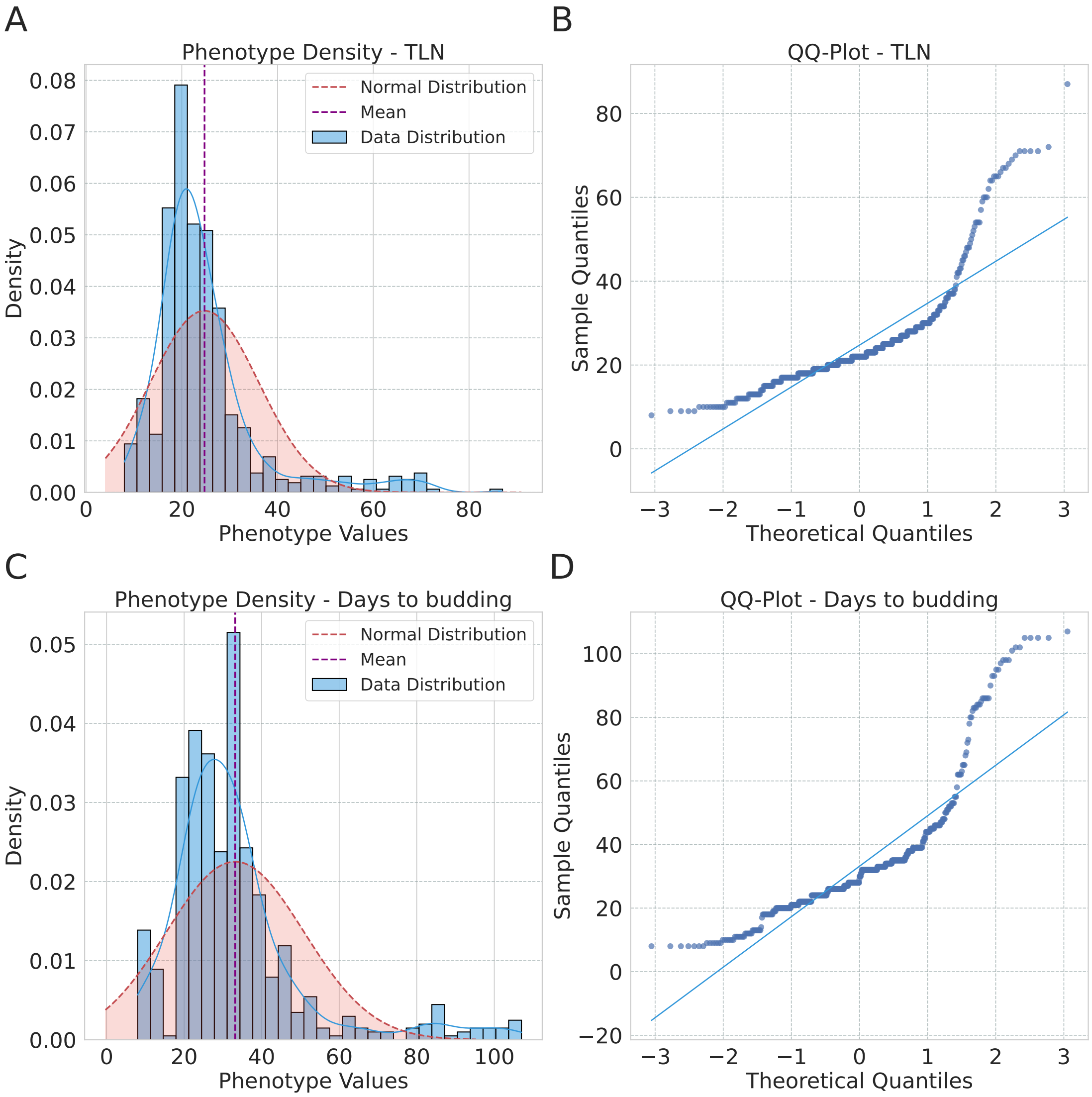


**Supplementary Figure 4: Phenotype distributions plotted as density and QQ-plots:** (A) and (C) phenotype density plot including fitted Gaussian for the phenotypes TLN (Shapiro-Wilk p-value: 6.22e-28) and Days to budding (Shapiro-Wilk p-value: 5.40e-27), respectively. (B) and (D) QQ-plots for phenotypes TLN and Days to budding.

# 4 Supplementary Results

### Phenotype: DTF

| | | | |
|---|---|---|---|
| Number of samples: | 586 | permGWAS2 Hits: | 6 |
| Number of SNPs: | 2681219 | permGWAS Hits: | 2 |
| Estimated heritability: | 9.99955E-1 | Bonferroni Hits: | 7 |

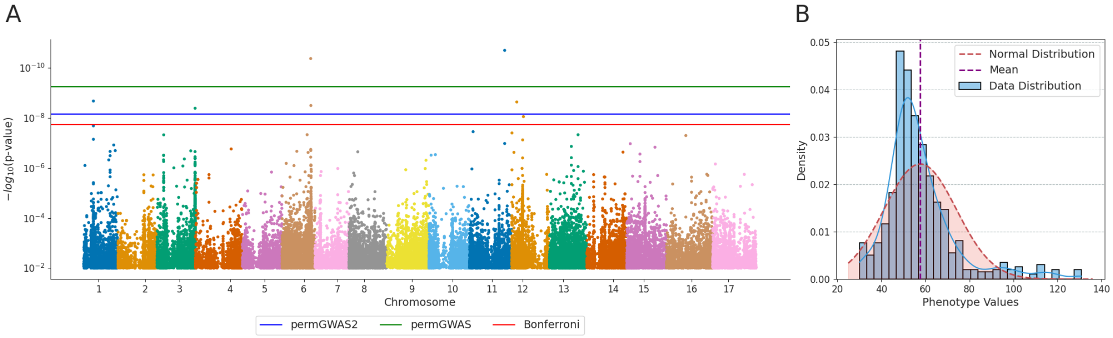


**Supplementary Figure 5: Manhattan and density plot of DTF:** (A) Manhattan plot with Bonferroni and two permutation-based thresholds. (B) Phenotypic distribution (Shapiro-Wilk p-value: 1.12930E-23)

**Supplementary Table 2: Significant Associations of DTF:** All significant associations found by at least one of the three thresholds permGWAS2 (perm2), permGWAS (perm), and Bonferroni (bonf). The columns show the chromosome, position and p-value of each hit. Additionally, the closest gene including the distance to the gene are shown. The last column contains the threshold(s) with respect to which the SNP was deemed significant.

| Chromosome | Position | P-value | Gene | Distance | Threshold |
|---|---|---|---|---|---|
| Ha412HOChr01 | 45111025 | 2.09123E-9 | Ha412HOChr01g0010021 | 0 | perm2,bonf |
| Ha412HOChr03 | 181204493 | 4.05164E-9 | Ha412HOChr03g0144601 | 25 | perm2,bonf |
| Ha412HOChr06 | 135718758 | 4.19370E-11 | Ha412HOChr06g0281751 | 23744 | perm2,perm,bonf |
| Ha412HOChr06 | 136571815 | 3.15252E-9 | Ha412HOChr06g0281881 | 16387 | perm2,bonf |
| Ha412HOChr11 | 165672035 | 1.96884E-11 | Ha412HOChr11g0520191 | 166692 | perm2,perm,bonf |
| Ha412HOChr12 | 23872475 | 2.27057E-9 | Ha412HOChr12g0545581 | 0 | perm2,bonf |
| Ha412HOChr12 | 55053233 | 8.75770E-9 | Ha412HOChr12g0555021 | 0 | bonf |

### Phenotype: Days to budding

| | | | |
|---|---|---|---|
| Number of samples: | 612 | `permGWAS2` Hits: | 237 |
| Number of SNPs: | 2673034 | `permGWAS` Hits: | 50 |
| Estimated heritability: | 9.99955E-1 | Bonferroni Hits: | 84 |

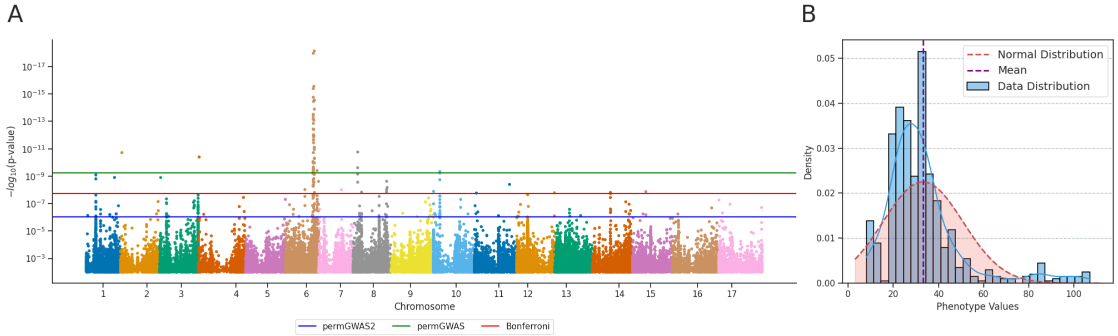


**Supplementary Figure 6: Manhattan and density plot of Days to budding:** (A) Manhattan plot with Bonferroni and two permutation-based thresholds. (B) Phenotypic distribution (Shapiro-Wilk p-value: 5.39959E-27)

**Supplementary Table 3: Significant Associations of Days to budding:** Significant associations within or close to a gene (distance $\leq 200$ bp) found by at least one of the three thresholds `permGWAS2` (perm2), `permGWAS` (perm), and Bonferroni (bonf). The columns show the chromosome, position and p-value of each hit. Additionally, the closest gene including the distance to the gene are shown. The last column contains the threshold(s) with respect to which the SNP was deemed significant.

| Chromosome | Position | P-value | Gene | Distance | Threshold |
|---|---|---|---|---|---|
| Ha412HOChr01 | 7131168 | 7.29682E-7 | Ha412HOChr01g0003121 | 0 | perm2 |
| Ha412HOChr01 | 45110583 | 8.09230E-10 | Ha412HOChr01g0010021 | 0 | perm2,bonf |
| Ha412HOChr01 | 45111025 | 1.56130E-9 | Ha412HOChr01g0010021 | 0 | perm2,bonf |
| Ha412HOChr01 | 45112167 | 6.77069E-8 | Ha412HOChr01g0010021 | 0 | perm2 |
| Ha412HOChr01 | 45112322 | 2.38241E-8 | Ha412HOChr01g0010021 | 0 | perm2 |
| Ha412HOChr01 | 45164894 | 1.68438E-7 | Ha412HOChr01g0010031 | 0 | perm2 |
| Ha412HOChr01 | 45178177 | 2.50135E-7 | Ha412HOChr01g0010031 | 0 | perm2 |
| Ha412HOChr01 | 132685321 | 1.24081E-9 | Ha412HOChr01g0034671 | 0 | perm2,bonf |
| Ha412HOChr01 | 140268828 | 5.61429E-7 | Ha412HOChr01g0037751 | 0 | perm2 |
| Ha412HOChr01 | 140268829 | 5.61429E-7 | Ha412HOChr01g0037751 | 0 | perm2 |
| Ha412HOChr01 | 151061648 | 1.44432E-7 | Ha412HOChr01g0043471 | 0 | perm2 |
| Ha412HOChr03 | 33389125 | 6.25023E-7 | Ha412HOChr03g0100731 | 0 | perm2 |
| Ha412HOChr03 | 42467534 | 8.36531E-7 | Ha412HOChr03g0102261 | 0 | perm2 |
| Ha412HOChr03 | 159296423 | 8.07028E-8 | Ha412HOChr03g0132801 | 0 | perm2 |
| Ha412HOChr03 | 181203221 | 1.20297E-7 | Ha412HOChr03g0144591 | 0 | perm2 |
| Ha412HOChr03 | 181204391 | 4.33568E-7 | Ha412HOChr03g0144601 | 127 | perm2 |
| Ha412HOChr03 | 181204493 | 2.39922E-8 | Ha412HOChr03g0144601 | 25 | perm2 |
| Ha412HOChr03 | 181293779 | 4.08803E-8 | Ha412HOChr03g0144781 | 135 | perm2 |
| Ha412HOChr03 | 181301933 | 4.66826E-7 | Ha412HOChr03g0144791 | 0 | perm2 |
| Ha412HOChr04 | 4467336 | 3.99077E-11 | Ha412HOChr04g0147001 | 0 | perm2,perm,bonf |
| Ha412HOChr04 | 212118212 | 3.53739E-8 | Ha412HOChr04g0194171 | 0 | perm2 |
| Ha412HOChr06 | 36559957 | 6.15009E-7 | Ha412HOChr06g0261111 | 0 | perm2 |

| | | | | | |
|---|---|---|---|---|---|
| Ha412HOChr06 | 130730537 | 4.05747E-16 | Ha412HOChr06g0281381 | 0 | perm2,perm,bonf |
| Ha412HOChr07 | 4550778 | 2.56840E-7 | Ha412HOChr07g0288531 | 0 | perm2 |
| Ha412HOChr07 | 107311931 | 9.92642E-9 | Ha412HOChr07g0305951 | 0 | perm2,bonf |
| Ha412HOChr08 | 21801953 | 6.95943E-10 | Ha412HOChr08g0337821 | 0 | perm2,bonf |
| Ha412HOChr08 | 21801975 | 2.74263E-9 | Ha412HOChr08g0337821 | 0 | perm2,bonf |
| Ha412HOChr08 | 30819055 | 1.46193E-7 | Ha412HOChr08g0341431 | 0 | perm2 |
| Ha412HOChr08 | 30819520 | 6.86556E-7 | Ha412HOChr08g0341431 | 0 | perm2 |
| Ha412HOChr08 | 157493582 | 2.35796E-9 | Ha412HOChr08g0368891 | 0 | perm2,bonf |
| Ha412HOChr08 | 159909069 | 6.68386E-9 | Ha412HOChr08g0369371 | 0 | perm2,bonf |
| Ha412HOChr09 | 183780555 | 1.24722E-7 | Ha412HOChr09g0420451 | 0 | perm2 |
| Ha412HOChr09 | 186015300 | 3.51418E-7 | Ha412HOChr09g0421401 | 0 | perm2 |
| Ha412HOChr09 | 188718842 | 2.62250E-7 | Ha412HOChr09g0422841 | 0 | perm2 |
| Ha412HOChr09 | 196164088 | 7.50750E-7 | Ha412HOChr09g0427921 | 0 | perm2 |
| Ha412HOChr10 | 703627 | 1.03463E-7 | Ha412HOChr10g0430511 | 0 | perm2 |
| Ha412HOChr10 | 9666517 | 6.51226E-7 | Ha412HOChr10g0433571 | 0 | perm2 |
| Ha412HOChr10 | 161487692 | 5.63304E-7 | Ha412HOChr10g0465881 | 0 | perm2 |
| Ha412HOChr11 | 8528165 | 5.38649E-7 | Ha412HOChr11g0483171 | 0 | perm2 |
| Ha412HOChr11 | 16945744 | 4.04424E-7 | Ha412HOChr11g0487001 | 0 | perm2 |
| Ha412HOChr12 | 10328784 | 4.16258E-7 | Ha412HOChr12g0539451 | 0 | perm2 |
| Ha412HOChr12 | 55053233 | 1.60252E-7 | Ha412HOChr12g0555021 | 0 | perm2 |
| Ha412HOChr12 | 177096137 | 1.61900E-8 | Ha412HOChr12g0580941 | 0 | perm2,bonf |
| Ha412HOChr13 | 69539362 | 4.78362E-7 | Ha412HOChr13g0596391 | 0 | perm2 |
| Ha412HOChr14 | 86395740 | 4.58879E-7 | Ha412HOChr14g0654311 | 0 | perm2 |
| Ha412HOChr14 | 86395768 | 3.62149E-8 | Ha412HOChr14g0654311 | 0 | perm2 |
| Ha412HOChr14 | 173882115 | 5.74498E-7 | Ha412HOChr14g0682631 | 0 | perm2 |
| Ha412HOChr14 | 173908348 | 1.10659E-7 | Ha412HOChr14g0682701 | 0 | perm2 |
| Ha412HOChr14 | 175514873 | 5.64836E-7 | Ha412HOChr14g0683571 | 0 | perm2 |
| Ha412HOChr14 | 181340146 | 3.20002E-7 | Ha412HOChr14g0687481 | 0 | perm2 |
| Ha412HOChr15 | 49160405 | 5.67811E-7 | Ha412HOChr15g0713071 | 0 | perm2 |
| Ha412HOChr17 | 1633300 | 5.52021E-8 | Ha412HOChr17g0804731 | 0 | perm2 |
| Ha412HOChr17 | 18025449 | 4.80208E-7 | Ha412HOChr17g0813621 | 0 | perm2 |
| Ha412HOChr17 | 201163759 | 1.93311E-7 | Ha412HOChr17g0856591 | 0 | perm2 |

**Phenotype: Distance of first branching from ground**

| | | | |
|---|---|---|---|
| Number of samples: | 602 | `permGWAS2` Hits: | 3 |
| Number of SNPs: | 2671483 | `permGWAS` Hits: | 3 |
| Estimated heritability: | 9.15197E-1 | Bonferroni Hits: | 3 |

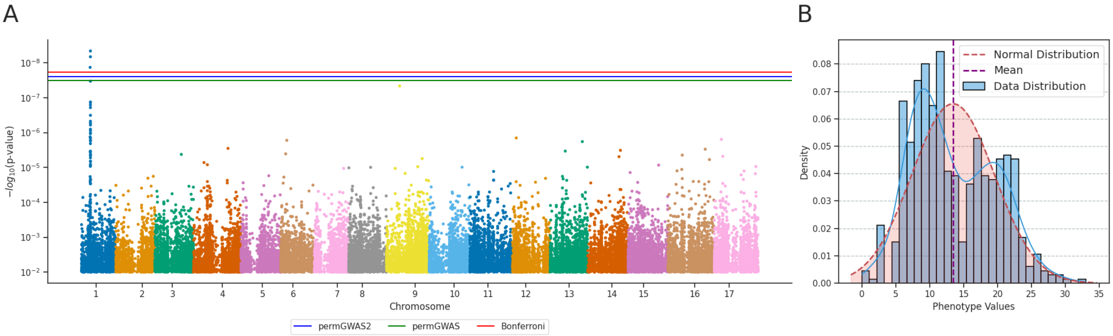


**Supplementary Figure 7: Manhattan and density plot of Distance of first branching from ground:** (A) Manhattan plot with Bonferroni and two permutation-based thresholds. (B) Phenotypic distribution (Shapiro-Wilk p-value: 3.04134E-10)

**Supplementary Table 4: Significant Associations of Distance of first branching from ground:** All significant associations found by at least one of the three thresholds `permGWAS2` (perm2), `permGWAS` (perm), and Bonferroni (bonf). The columns show the chromosome, position and p-value of each hit. Additionally, the closest gene including the distance to the gene are shown. The last column contains the threshold(s) with respect to which the SNP was deemed significant.

| Chromosome | Position | P-value | Gene | Distance | Threshold |
|---|---|---|---|---|---|
| Ha412HOChr01 | 40874604 | 1.34771E-8 | Ha412HOChr01g0009251 | 162829 | perm2,perm,bonf |
| Ha412HOChr01 | 42294278 | 4.57420E-9 | Ha412HOChr01g0009401 | 126653 | perm2,perm,bonf |
| Ha412HOChr01 | 42299923 | 6.70299E-9 | Ha412HOChr01g0009401 | 132298 | perm2,perm,bonf |

## Phenotype: LIR

| | | | |
|---|---|---|---|
| Number of samples: | 604 | `permGWAS2` Hits: | 8 |
| Number of SNPs: | 2677331 | `permGWAS` Hits: | 5 |
| Estimated heritability: | 7.44106E-1 | Bonferroni Hits: | 21 |

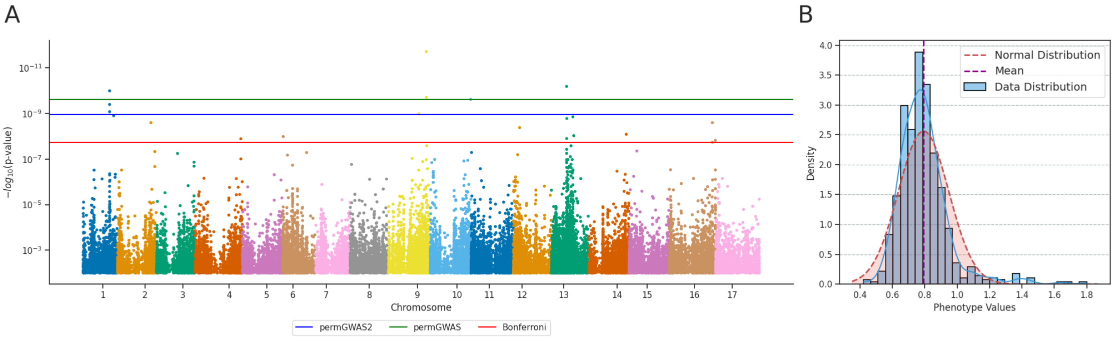


**Supplementary Figure 8: Manhattan and density plot of LIR:** (A) Manhattan plot with Bonferroni and two permutation-based thresholds. (B) Phenotypic distribution (Shapiro-Wilk p-value: 9.57086E-23)

**Supplementary Table 5: Significant Associations of LIR:** All significant associations found by at least one of the three thresholds `permGWAS2` (perm2), `permGWAS` (perm), and Bonferroni (bonf). The columns show the chromosome, position and p-value of each hit. Additionally, the closest gene including the distance to the gene are shown. The last column contains the threshold(s) with respect to which the SNP was deemed significant.

| Chromosome | Position | P-value | Gene | Distance | Threshold |
|---|---|---|---|---|---|
| Ha412HOChr01 | 124116926 | 4.05670E-10 | Ha412HOChr01g0031661 | 33132 | perm2,bonf |
| Ha412HOChr01 | 124117180 | 1.02720E-10 | Ha412HOChr01g0031661 | 32878 | perm2,perm,bonf |
| Ha412HOChr01 | 124151993 | 8.47286E-10 | Ha412HOChr01g0031661 | 0 | perm2,bonf |
| Ha412HOChr01 | 143235204 | 1.26103E-9 | Ha412HOChr01g0039351 | 0 | bonf |
| Ha412HOChr02 | 158884005 | 2.55500E-9 | Ha412HOChr02g0081531 | 1373 | bonf |
| Ha412HOChr04 | 214170232 | 1.30212E-8 | Ha412HOChr04g0195421 | 4297 | bonf |
| Ha412HOChr06 | 4234410 | 1.03756E-8 | Ha412HOChr06g0247791 | 0 | bonf |
| Ha412HOChr09 | 146135316 | 1.05470E-9 | Ha412HOChr09g0405881 | 16487 | perm2,bonf |
| Ha412HOChr09 | 180697542 | 2.01232E-10 | Ha412HOChr09g0418941 | 5893 | perm2,perm,bonf |
| Ha412HOChr09 | 180697556 | 1.94574E-12 | Ha412HOChr09g0418941 | 5879 | perm2,perm,bonf |
| Ha412HOChr10 | 188909962 | 2.41232E-10 | Ha412HOChr10g0477251 | 3014 | perm2,perm,bonf |
| Ha412HOChr12 | 28829950 | 4.20670E-9 | Ha412HOChr12g0547561 | 40353 | bonf |
| Ha412HOChr13 | 71865495 | 6.55280E-11 | Ha412HOChr13g0597091 | 5004 | perm2,perm,bonf |
| Ha412HOChr13 | 72067833 | 1.27538E-8 | Ha412HOChr13g0597181 | 31562 | bonf |
| Ha412HOChr13 | 72579067 | 1.67537E-9 | Ha412HOChr13g0597311 | 842 | bonf |
| Ha412HOChr13 | 101236505 | 1.42248E-9 | Ha412HOChr13g0607401 | 60621 | bonf |
| Ha412HOChr13 | 105145999 | 9.46501E-9 | Ha412HOChr13g0608391 | 0 | bonf |
| Ha412HOChr14 | 175611402 | 8.21044E-9 | Ha412HOChr14g0683671 | 1854 | bonf |
| Ha412HOChr16 | 203519793 | 2.54764E-9 | Ha412HOChr16g0799351 | 44366 | bonf |
| Ha412HOChr16 | 203531980 | 1.83253E-8 | Ha412HOChr16g0799351 | 32179 | bonf |
| Ha412HOChr16 | 217773720 | 1.52857E-8 | Ha412HOChr16g0803941 | 11038 | bonf |

### Phenotype: Leaf C Nratio

| | | | |
|---|---|---|---|
| Number of samples: | 606 | `permGWAS2` Hits: | 0 |
| Number of SNPs: | 2681149 | `permGWAS` Hits: | 0 |
| Estimated heritability: | 9.26682E-1 | Bonferroni Hits: | 1 |

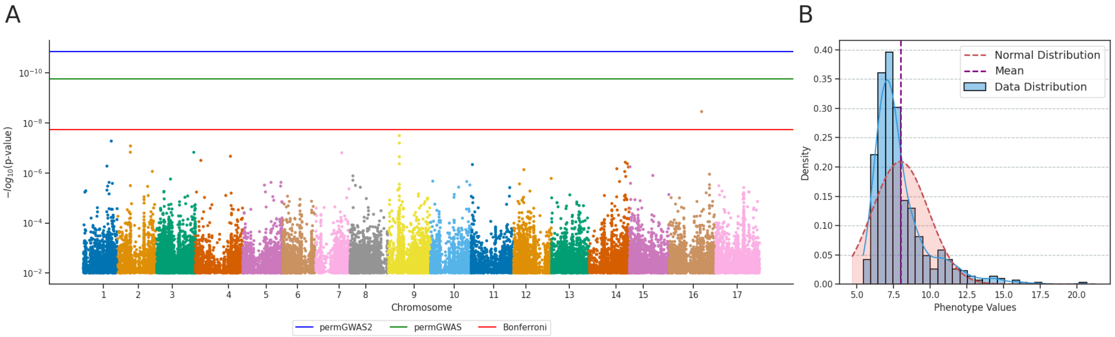


**Supplementary Figure 9: Manhattan and density plot of Leaf C Nratio:** (A) Manhattan plot with Bonferroni and two permutation-based thresholds. (B) Phenotypic distribution (Shapiro-Wilk p-value: 7.97533E-27)

**Supplementary Table 6: Significant Associations of Leaf C Nratio:** All significant associations found by at least one of the three thresholds `permGWAS2` (perm2), `permGWAS` (perm), and Bonferroni (bonf). The columns show the chromosome, position and p-value of each hit. Additionally, the closest gene including the distance to the gene are shown. The last column contains the threshold(s) with respect to which the SNP was deemed significant.

| Chromosome | Position | P-value | Gene | Distance | Threshold |
|---|---|---|---|---|---|
| Ha412HOChr16 | 152811922 | 3.49114E-9 | Ha412HOChr16g0782101 | 3 | bonf |

## Phenotype: Leaf Distal eccentricity[ANN rm218P195]

| | | | |
|---|---|---|---|
| Number of samples: | 599 | permGWAS2 Hits: | 0 |
| Number of SNPs: | 2688886 | permGWAS Hits: | 0 |
| Estimated heritability: | 1.12296E-1 | Bonferroni Hits: | 7 |

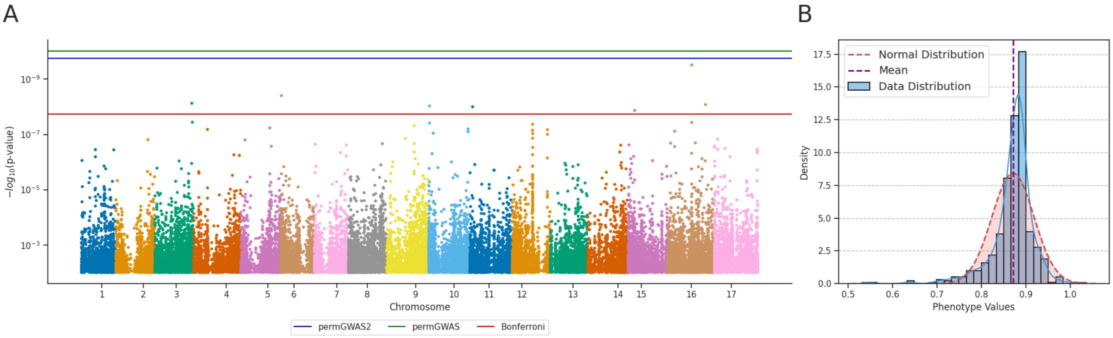


**Supplementary Figure 10: Manhattan and density plot of Leaf Distal eccentricity[ANN rm218P195]:** (A) Manhattan plot with Bonferroni and two permutation-based thresholds. (B) Phenotypic distribution (Shapiro-Wilk p-value: 8.77871E-25)

**Supplementary Table 7: Significant Associations of Leaf Distal eccentricity[ANN rm218P195]:** All significant associations found by at least one of the three thresholds permGWAS2 (perm2), permGWAS (perm), and Bonferroni (bonf). The columns show the chromosome, position and p-value of each hit. Additionally, the closest gene including the distance to the gene are shown. The last column contains the threshold(s) with respect to which the SNP was deemed significant.

| Chromosome | Position | P-value | Gene | Distance | Threshold |
|---|---|---|---|---|---|
| Ha412HOChr03 | 174095016 | 7.44881E-9 | Ha412HOChr03g0139431 | 0 | bonf |
| Ha412HOChr06 | 3533967 | 3.93447E-9 | Ha412HOChr06g0247051 | 0 | bonf |
| Ha412HOChr10 | 2285874 | 9.33478E-9 | Ha412HOChr10g0430971 | 3561 | bonf |
| Ha412HOChr11 | 13389989 | 1.00358E-8 | Ha412HOChr11g0485701 | 0 | bonf |
| Ha412HOChr15 | 31048243 | 1.35731E-8 | Ha412HOChr15g0707391 | 0 | bonf |
| Ha412HOChr16 | 112791737 | 3.04655E-10 | Ha412HOChr16g0772631 | 50199 | bonf |
| Ha412HOChr16 | 177261998 | 8.32572E-9 | Ha412HOChr16g0788831 | 17186 | bonf |

## Phenotype: Leaf circular[ANN rm218P189]

| | | | |
|---|---|---|---|
| Number of samples: | 599 | permGWAS2 Hits: | 1 |
| Number of SNPs: | 2688886 | permGWAS Hits: | 1 |
| Estimated heritability: | 8.11798E-1 | Bonferroni Hits: | 1 |

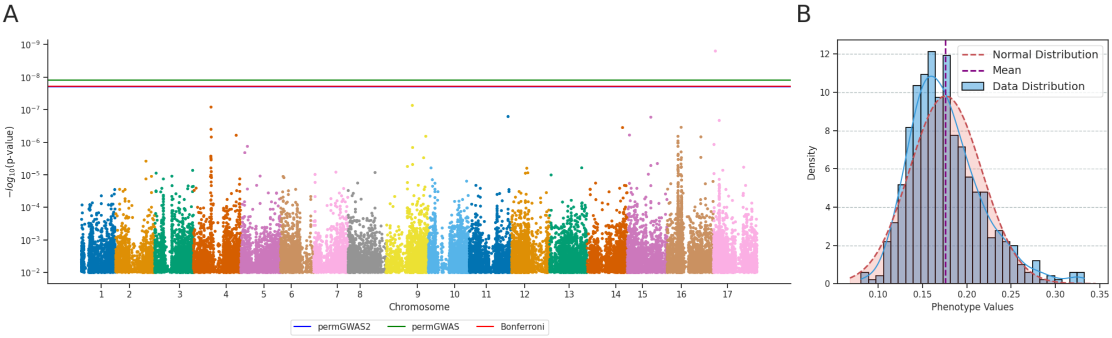


**Supplementary Figure 11: Manhattan and density plot of Leaf circular[ANN rm218P189]:** (A) Manhattan plot with Bonferroni and two permutation-based thresholds. (B) Phenotypic distribution (Shapiro-Wilk p-value: 2.27289E-12)

**Supplementary Table 8: Significant Associations of Leaf circular[ANN rm218P189]:** All significant associations found by at least one of the three thresholds permGWAS2 (perm2), permGWAS (perm), and Bonferroni (bonf). The columns show the chromosome, position and p-value of each hit. Additionally, the closest gene including the distance to the gene are shown. The last column contains the threshold(s) with respect to which the SNP was deemed significant.

| Chromosome | Position | P-value | Gene | Distance | Threshold |
|---|---|---|---|---|---|
| Ha412HOChr17 | 9116266 | 1.57428E-9 | Ha412HOChr17g0808701 | 0 | perm2,perm,bonf |

### Phenotype: Leaf curvedHeight maxWidth[ANN rm218P143]

| | | | |
|---|---|---|---|
| Number of samples: | 599 | permGWAS2 Hits: | 1 |
| Number of SNPs: | 2688886 | permGWAS Hits: | 0 |
| Estimated heritability: | 8.59637E-1 | Bonferroni Hits: | 3 |

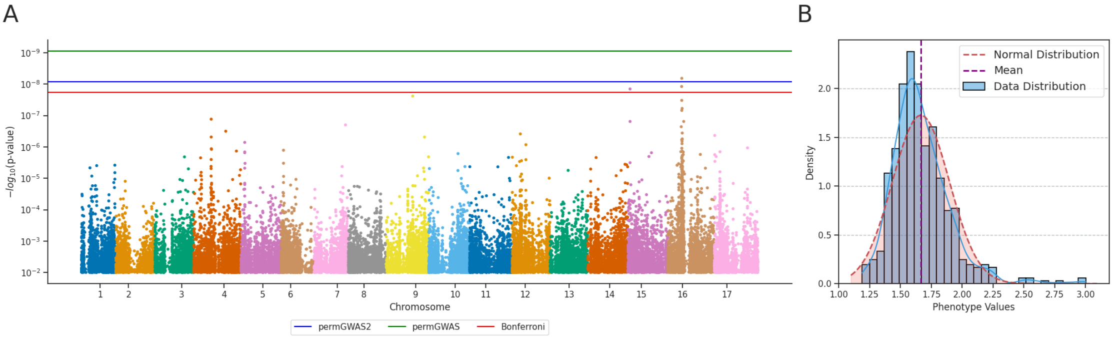


**Supplementary Figure 12: Manhattan and density plot of Leaf curvedHeight maxWidth[ANN rm218P143]:** (A) Manhattan plot with Bonferroni and two permutation-based thresholds. (B) Phenotypic distribution (Shapiro-Wilk p-value: 5.33479E-18)

**Supplementary Table 9: Significant Associations of Leaf curvedHeight maxWidth[ANN rm218P143]:** All significant associations found by at least one of the three thresholds `permGWAS2` (perm2), `permGWAS` (perm), and Bonferroni (bonf). The columns show the chromosome, position and p-value of each hit. Additionally, the closest gene including the distance to the gene are shown. The last column contains the threshold(s) with respect to which the SNP was deemed significant.

| Chromosome | Position | P-value | Gene | Distance | Threshold |
|---|---|---|---|---|---|
| Ha412HOChr15 | 9551550 | 1.42838E-8 | Ha412HOChr15g0699071 | 12042 | bonf |
| Ha412HOChr16 | 65091331 | 1.19377E-8 | Ha412HOChr16g0764961 | 0 | bonf |
| Ha412HOChr16 | 65929716 | 6.54801E-9 | Ha412HOChr16g0765031 | 31230 | perm2,bonf |

**Phenotype: Leaf eccentricity[ANN rm218P193]**

| | | | |
|---|---|---|---|
| Number of samples: | 599 | `permGWAS2` Hits: | 0 |
| Number of SNPs: | 2688886 | `permGWAS` Hits: | 0 |
| Estimated heritability: | 1.94421E-1 | Bonferroni Hits: | 1 |

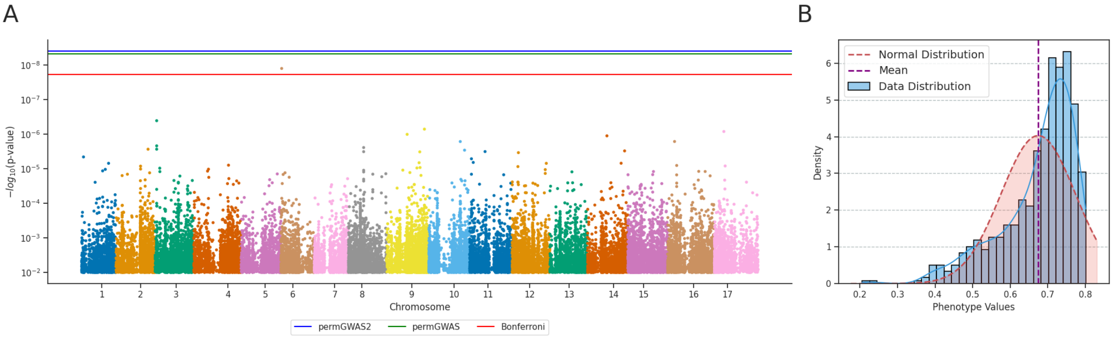


**Supplementary Figure 13: Manhattan and density plot of Leaf eccentricity[ANN rm218P193]:** (A) Manhattan plot with Bonferroni and two permutation-based thresholds. (B) Phenotypic distribution (Shapiro-Wilk p-value: 6.27907E-21)

**Supplementary Table 10: Significant Associations of Leaf eccentricity[ANN rm218P193]:** All significant associations found by at least one of the three thresholds `permGWAS2` (perm2), `permGWAS` (perm), and Bonferroni (bonf). The columns show the chromosome, position and p-value of each hit. Additionally, the closest gene including the distance to the gene are shown. The last column contains the threshold(s) with respect to which the SNP was deemed significant.

| Chromosome | Position | P-value | Gene | Distance | Threshold |
|---|---|---|---|---|---|
| Ha412HOChr06 | 3533967 | 1.24241E-8 | Ha412HOChr06g0247051 | 0 | bonf |

**Phenotype: Leaf height mid-width[ANN rm218P140]**

| | | | |
|---|---|---|---|
| Number of samples: | 599 | permGWAS2 Hits: | 0 |
| Number of SNPs: | 2688886 | permGWAS Hits: | 1 |
| Estimated heritability: | 7.02470E-1 | Bonferroni Hits: | 0 |

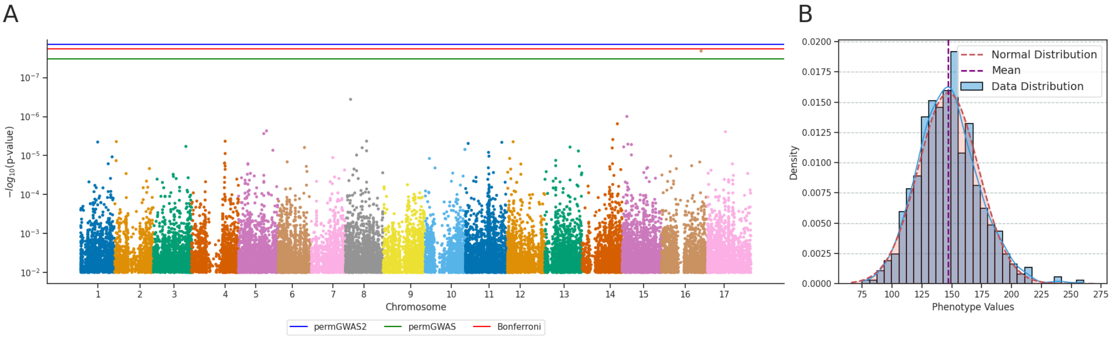


**Supplementary Figure 14: Manhattan and density plot of Leaf height mid-width[ANN rm218P140]:** (A) Manhattan plot with Bonferroni and two permutation-based thresholds. (B) Phenotypic distribution (Shapiro-Wilk p-value: 3.14576E-4)

**Supplementary Table 11: Significant Associations of Leaf height mid-width[ANN rm218P140]:** All significant associations found by at least one of the three thresholds permGWAS2 (perm2), permGWAS (perm), and Bonferroni (bonf). The columns show the chromosome, position and p-value of each hit. Additionally, the closest gene including the distance to the gene are shown. The last column contains the threshold(s) with respect to which the SNP was deemed significant.

| Chromosome | Position | P-value | Gene | Distance | Threshold |
|---|---|---|---|---|---|
| Ha412HOChr16 | 188412525 | 2.08585E-8 | Ha412HOChr16g0792511 | 0 | perm |

## Phenotype: Leaf shape index external II[ANN rm218P186]

| | | | |
|---|---|---|---|
| Number of samples: | 599 | permGWAS2 Hits: | 3 |
| Number of SNPs: | 2688886 | permGWAS Hits: | 2 |
| Estimated heritability: | 7.26358E-1 | Bonferroni Hits: | 3 |

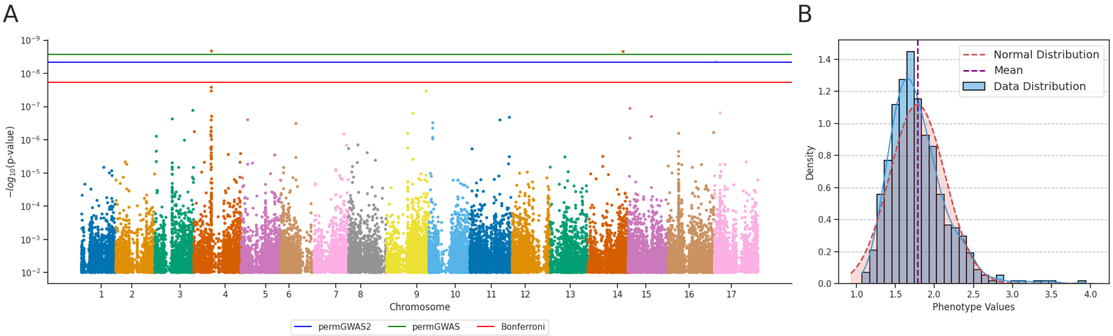


**Supplementary Figure 15: Manhattan and density plot of Leaf shape index external II[ANN rm218P186]:** (A) Manhattan plot with Bonferroni and two permutation-based thresholds. (B) Phenotypic distribution (Shapiro-Wilk p-value: 1.12985E-16)

**Supplementary Table 12: Significant Associations of Leaf shape index external II[ANN rm218P186]:** All significant associations found by at least one of the three thresholds permGWAS2 (perm2), permGWAS (perm), and Bonferroni (bonf). The columns show the chromosome, position and p-value of each hit. Additionally, the closest gene including the distance to the gene are shown. The last column contains the threshold(s) with respect to which the SNP was deemed significant.

| Chromosome | Position | P-value | Gene | Distance | Threshold |
|---|---|---|---|---|---|
| Ha412HOChr04 | 84015571 | 2.11752E-9 | Ha412HOChr04g0167121 | 482 | perm2,perm,bonf |
| Ha412HOChr14 | 166525616 | 2.19311E-9 | Ha412HOChr14g0678931 | 3519 | perm2,perm,bonf |
| Ha412HOChr17 | 9116266 | 4.40611E-9 | Ha412HOChr17g0808701 | 0 | perm2,bonf |

## Phenotype: Leaf shape index external I[ANN rm218P185]

| | | | |
|---|---|---|---|
| Number of samples: | 599 | `permGWAS2` Hits: | 2 |
| Number of SNPs: | 2688886 | `permGWAS` Hits: | 0 |
| Estimated heritability: | 8.79787E-1 | Bonferroni Hits: | 4 |

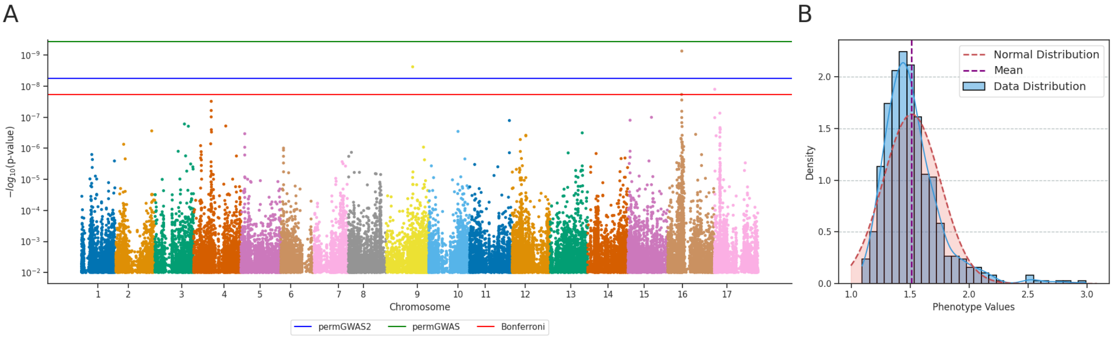


**Supplementary Figure 16: Manhattan and density plot of Leaf shape index external I[ANN rm218P185]:** (A) Manhattan plot with Bonferroni and two permutation-based thresholds. (B) Phenotypic distribution (Shapiro-Wilk p-value: 5.64111E-22)

**Supplementary Table 13: Significant Associations of Leaf shape index external I[ANN rm218P185]:** All significant associations found by at least one of the three thresholds `permGWAS2` (perm2), `permGWAS` (perm), and Bonferroni (bonf). The columns show the chromosome, position and p-value of each hit. Additionally, the closest gene including the distance to the gene are shown. The last column contains the threshold(s) with respect to which the SNP was deemed significant.

| Chromosome | Position | P-value | Gene | Distance | Threshold |
|---|---|---|---|---|---|
| Ha412HOChr09 | 124355109 | 2.38632E-9 | Ha412HOChr09g0397831 | 4158 | perm2,bonf |
| Ha412HOChr16 | 65091331 | 1.84823E-8 | Ha412HOChr16g0764961 | 0 | bonf |
| Ha412HOChr16 | 65929716 | 7.41722E-10 | Ha412HOChr16g0765031 | 31230 | perm2,bonf |
| Ha412HOChr17 | 2652319 | 1.25962E-8 | Ha412HOChr17g0805341 | 8424 | bonf |

**Phenotype: Leaf shape index internal[ANN rm218P196]**

| | | | |
|---|---|---|---|
| Number of samples: | 599 | permGWAS2 Hits: | 0 |
| Number of SNPs: | 2688886 | permGWAS Hits: | 0 |
| Estimated heritability: | 4.53363E-1 | Bonferroni Hits: | 1 |

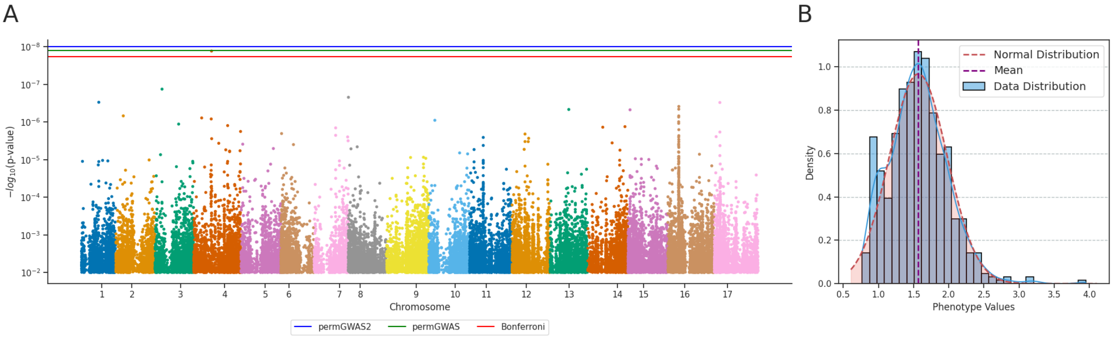


**Supplementary Figure 17: Manhattan and density plot of Leaf shape index internal[ANN rm218P196]:** (A) Manhattan plot with Bonferroni and two permutation-based thresholds. (B) Phenotypic distribution (Shapiro-Wilk p-value: 3.10460E-10)

**Supplementary Table 14: Significant Associations of Leaf shape index internal[ANN rm218P196]:** All significant associations found by at least one of the three thresholds permGWAS2 (perm2), permGWAS (perm), and Bonferroni (bonf). The columns show the chromosome, position and p-value of each hit. Additionally, the closest gene including the distance to the gene are shown. The last column contains the threshold(s) with respect to which the SNP was deemed significant.

| Chromosome | Position | P-value | Gene | Distance | Threshold |
|---|---|---|---|---|---|
| Ha412HOChr04 | 84015571 | 1.30257E-8 | Ha412HOChr04g0167121 | 482 | bonf |

## Phenotype: Leaf total C

| | | | |
|---|---|---|---|
| Number of samples: | 606 | `permGWAS2` Hits: | 0 |
| Number of SNPs: | 2681149 | `permGWAS` Hits: | 0 |
| Estimated heritability: | 5.54707E-1 | Bonferroni Hits: | 5 |

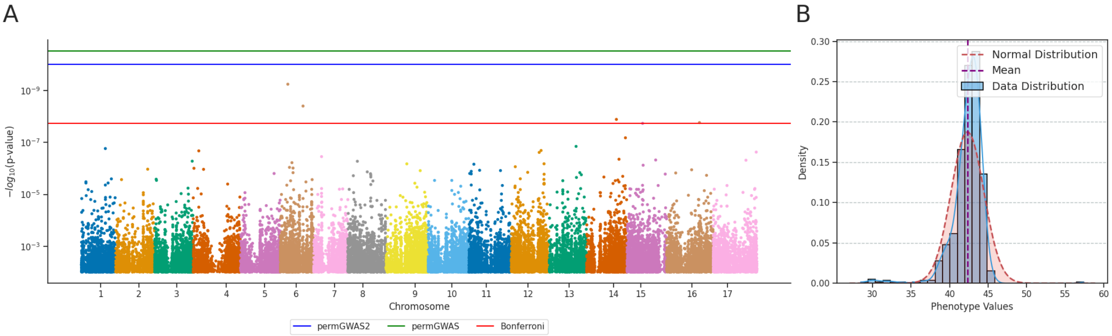


**Supplementary Figure 18: Manhattan and density plot of Leaf total C:** (A) Manhattan plot with Bonferroni and two permutation-based thresholds. (B) Phenotypic distribution (Shapiro-Wilk p-value: 1.03772E-28)

**Supplementary Table 15: Significant Associations of Leaf total C:** All significant associations found by at least one of the three thresholds `permGWAS2` (perm2), `permGWAS` (perm), and Bonferroni (bonf). The columns show the chromosome, position and p-value of each hit. Additionally, the closest gene including the distance to the gene are shown. The last column contains the threshold(s) with respect to which the SNP was deemed significant.

| Chromosome | Position | P-value | Gene | Distance | Threshold |
|---|---|---|---|---|---|
| Ha412HOChr06 | 35358089 | 5.62039E-10 | Ha412HOChr06g0260731 | 128252 | bonf |
| Ha412HOChr06 | 106598011 | 3.91381E-9 | Ha412HOChr06g0277841 | 1266 | bonf |
| Ha412HOChr14 | 140484458 | 1.28137E-8 | Ha412HOChr14g0669231 | 1908 | bonf |
| Ha412HOChr15 | 73564147 | 1.82103E-8 | Ha412HOChr15g0720611 | 47901 | bonf |
| Ha412HOChr16 | 154341301 | 1.69763E-8 | Ha412HOChr16g0782441 | 0 | bonf |

## Phenotype: Phyllaries width[ANN rm218P204]

| | | | |
|---|---|---|---|
| Number of samples: | 574 | permGWAS2 Hits: | 1 |
| Number of SNPs: | 2676237 | permGWAS Hits: | 0 |
| Estimated heritability: | 9.99955E-1 | Bonferroni Hits: | 0 |

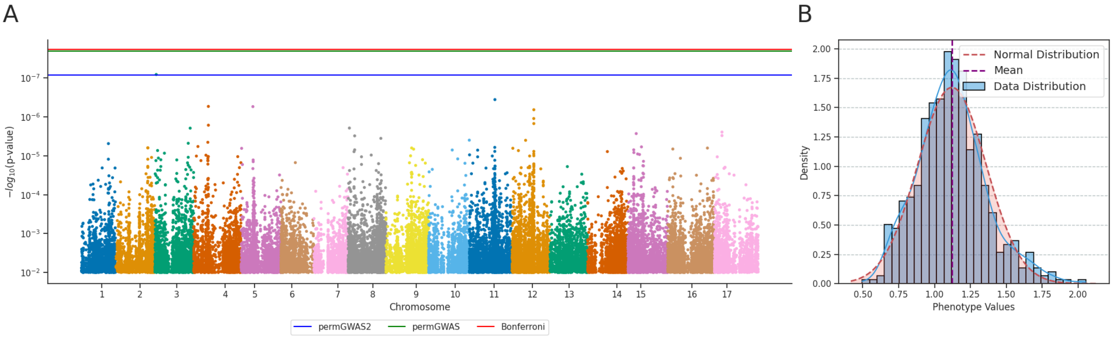


**Supplementary Figure 19: Manhattan and density plot of Phyllaries width[ANN rm218P204]:** (A) Manhattan plot with Bonferroni and two permutation-based thresholds. (B) Phenotypic distribution (Shapiro-Wilk p-value: 6.98668E-6)

**Supplementary Table 16: Significant Associations of Phyllaries width[ANN rm218P204]:** All significant associations found by at least one of the three thresholds permGWAS2 (perm2), permGWAS (perm), and Bonferroni (bonf). The columns show the chromosome, position and p-value of each hit. Additionally, the closest gene including the distance to the gene are shown. The last column contains the threshold(s) with respect to which the SNP was deemed significant.

| Chromosome | Position | P-value | Gene | Distance | Threshold |
|---|---|---|---|---|---|
| Ha412HOChr03 | 6609218 | 8.14627E-8 | Ha412HOChr03g0093891 | 233226 | perm2 |

## Phenotype: Plant height at flowering

| | | | |
|---|---|---|---|
| Number of samples: | 585 | permGWAS2 Hits: | 3 |
| Number of SNPs: | 2678757 | permGWAS Hits: | 1 |
| Estimated heritability: | 9.99955E-1 | Bonferroni Hits: | 2 |

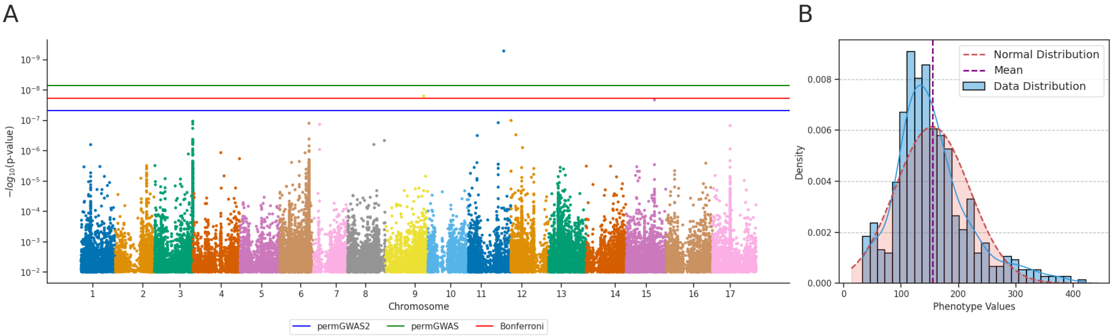


**Supplementary Figure 20: Manhattan and density plot of Plant height at flowering:** (A) Manhattan plot with Bonferroni and two permutation-based thresholds. (B) Phenotypic distribution (Shapiro-Wilk p-value: 4.19968E-15)

**Supplementary Table 17: Significant Associations of Plant height at flowering:** All significant associations found by at least one of the three thresholds permGWAS2 (perm2), permGWAS (perm), and Bonferroni (bonf). The columns show the chromosome, position and p-value of each hit. Additionally, the closest gene including the distance to the gene are shown. The last column contains the threshold(s) with respect to which the SNP was deemed significant.

| Chromosome | Position | P-value | Gene | Distance | Threshold |
|---|---|---|---|---|---|
| Ha412HOChr09 | 180416349 | 1.58676E-8 | Ha412HOChr09g0418821 | 28816 | perm2,bonf |
| Ha412HOChr11 | 165672035 | 5.17668E-10 | Ha412HOChr11g0520191 | 166692 | perm2,perm,bonf |
| Ha412HOChr15 | 132367715 | 2.11544E-8 | Ha412HOChr15g0735201 | 98823 | perm2 |

### Phenotype: Primary branches

| | | | |
|---|---|---|---|
| Number of samples: | 584 | permGWAS2 Hits: | 88 |
| Number of SNPs: | 2674184 | permGWAS Hits: | 29 |
| Estimated heritability: | 9.99955E-1 | Bonferroni Hits: | 58 |

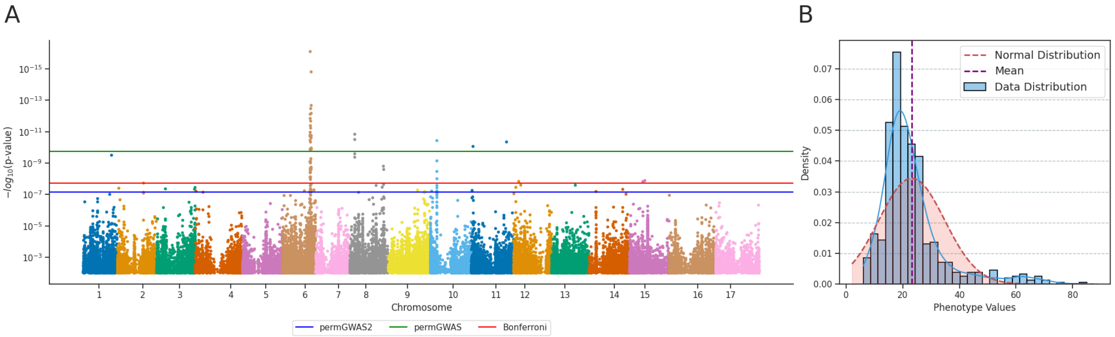


**Supplementary Figure 21: Manhattan and density plot of Primary branches:** (A) Manhattan plot with Bonferroni and two permutation-based thresholds. (B) Phenotypic distribution (Shapiro-Wilk p-value: 1.26855E-26)

**Supplementary Table 18: Significant Associations of Primary branches:** Significant associations within or close to a gene (distance $\leq$ 200 bp) found by at least one of the three thresholds permGWAS2 (perm2), permGWAS (perm), and Bonferroni (bonf). The columns show the chromosome, position and p-value of each hit. Additionally, the closest gene including the distance to the gene are shown. The last column contains the threshold(s) with respect to which the SNP was deemed significant.

| Chromosome | Position | P-value | Gene | Distance | Threshold |
|---|---|---|---|---|---|
| Ha412HOChr01 | 132685321 | 3.15103E-10 | Ha412HOChr01g0034671 | 0 | perm2,bonf |
| Ha412HOChr03 | 42465356 | 4.37112E-8 | Ha412HOChr03g0102261 | 0 | perm2 |
| Ha412HOChr03 | 179000456 | 4.63890E-8 | Ha412HOChr03g0143151 | 0 | perm2 |
| Ha412HOChr03 | 181293779 | 3.58981E-8 | Ha412HOChr03g0144781 | 135 | perm2 |
| Ha412HOChr04 | 4467336 | 6.32323E-8 | Ha412HOChr04g0147001 | 0 | perm2 |
| Ha412HOChr06 | 103428315 | 5.81813E-8 | Ha412HOChr06g0277141 | 0 | perm2 |
| Ha412HOChr06 | 130379263 | 3.44249E-11 | Ha412HOChr06g0281361 | 0 | perm2,perm,bonf |
| Ha412HOChr06 | 130730537 | 7.75376E-13 | Ha412HOChr06g0281381 | 0 | perm2,perm,bonf |
| Ha412HOChr08 | 21801953 | 3.16037E-11 | Ha412HOChr08g0337821 | 0 | perm2,perm,bonf |
| Ha412HOChr08 | 21801975 | 1.46073E-11 | Ha412HOChr08g0337821 | 0 | perm2,perm,bonf |
| Ha412HOChr08 | 150578765 | 3.43889E-8 | Ha412HOChr08g0368051 | 0 | perm2 |
| Ha412HOChr08 | 156178808 | 1.57986E-9 | Ha412HOChr08g0368721 | 0 | perm2,bonf |
| Ha412HOChr08 | 157493582 | 2.54172E-9 | Ha412HOChr08g0368891 | 0 | perm2,bonf |
| Ha412HOChr11 | 8528165 | 8.69874E-11 | Ha412HOChr11g0483171 | 0 | perm2,perm,bonf |
| Ha412HOChr12 | 10354847 | 3.50139E-8 | Ha412HOChr12g0539461 | 0 | perm2 |
| Ha412HOChr12 | 23872475 | 1.46120E-8 | Ha412HOChr12g0545581 | 0 | perm2,bonf |

## Phenotype: RGB proportion green[ANN rm218P199]

| | | | |
|---|---|---|---|
| Number of samples: | 596 | `permGWAS2` Hits: | 0 |
| Number of SNPs: | 2681642 | `permGWAS` Hits: | 0 |
| Estimated heritability: | 3.50942E-1 | Bonferroni Hits: | 2 |

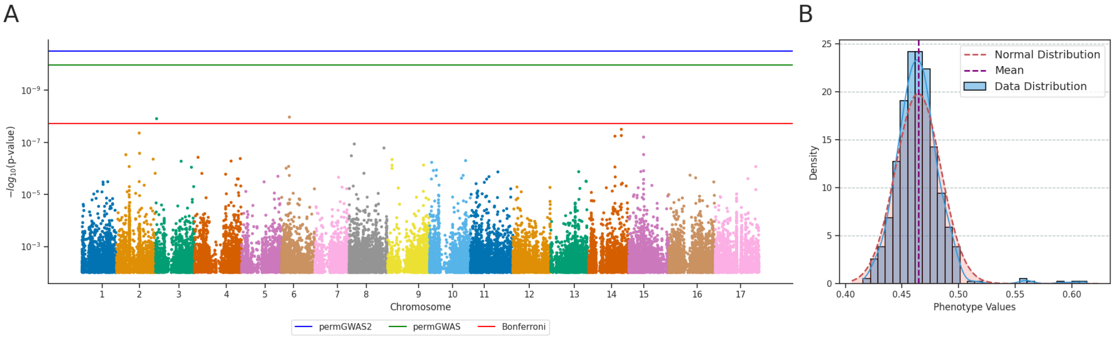


**Supplementary Figure 22: Manhattan and density plot of RGB proportion green[ANN rm218P199]:** (A) Manhattan plot with Bonferroni and two permutation-based thresholds. (B) Phenotypic distribution (Shapiro-Wilk p-value: 4.87988E-22)

**Supplementary Table 19: Significant Associations of RGB proportion green[ANN rm218P199]:** All significant associations found by at least one of the three thresholds `permGWAS2` (perm2), `permGWAS` (perm), and Bonferroni (bonf). The columns show the chromosome, position and p-value of each hit. Additionally, the closest gene including the distance to the gene are shown. The last column contains the threshold(s) with respect to which the SNP was deemed significant.

| Chromosome | Position | P-value | Gene | Distance | Threshold |
|---|---|---|---|---|---|
| Ha412HOChr03 | 5457435 | 1.23404E-8 | Ha412HOChr03g0093711 | 0 | bonf |
| Ha412HOChr06 | 37056163 | 1.07415E-8 | Ha412HOChr06g0261241 | 15594 | bonf |

### Phenotype: Seed distal eccentricity[ANN rm218P118]

| | | | |
|---|---|---|---|
| Number of samples: | 426 | permGWAS2 Hits: | 0 |
| Number of SNPs: | 2682419 | permGWAS Hits: | 0 |
| Estimated heritability: | 2.11277E-2 | Bonferroni Hits: | 1 |

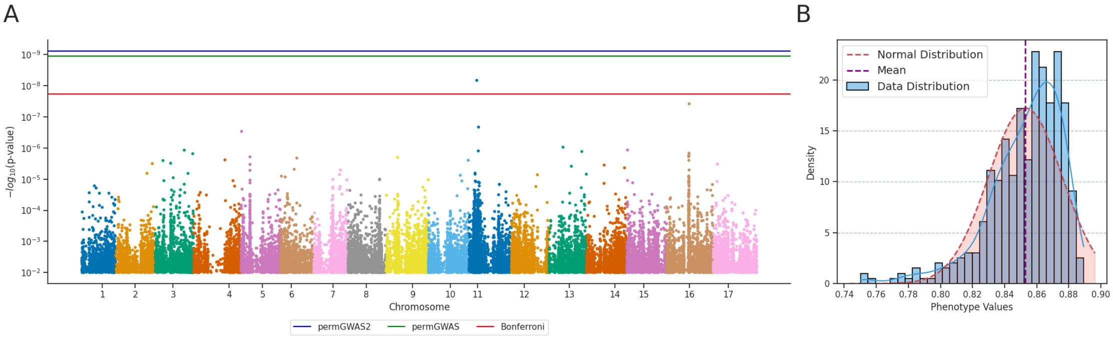


**Supplementary Figure 23: Manhattan and density plot of Seed distal eccentricity[ANN rm218P118]:** (A) Manhattan plot with Bonferroni and two permutation-based thresholds. (B) Phenotypic distribution (Shapiro-Wilk p-value: 1.18330E-15)

**Supplementary Table 20: Significant Associations of Seed distal eccentricity[ANN rm218P118]:** All significant associations found by at least one of the three thresholds permGWAS2 (perm2), permGWAS (perm), and Bonferroni (bonf). The columns show the chromosome, position and p-value of each hit. Additionally, the closest gene including the distance to the gene are shown. The last column contains the threshold(s) with respect to which the SNP was deemed significant.

| Chromosome | Position | P-value | Gene | Distance | Threshold |
|---|---|---|---|---|---|
| Ha412HOChr11 | 36373379 | 6.74398E-9 | Ha412HOChr11g0492621 | 2434 | bonf |

## Phenotype: Seed maximum width[ANN rm218P103]

| | | | |
|---|---|---|---|
| Number of samples: | 426 | permGWAS2 Hits: | 1 |
| Number of SNPs: | 2682419 | permGWAS Hits: | 0 |
| Estimated heritability: | 9.99955E-1 | Bonferroni Hits: | 0 |

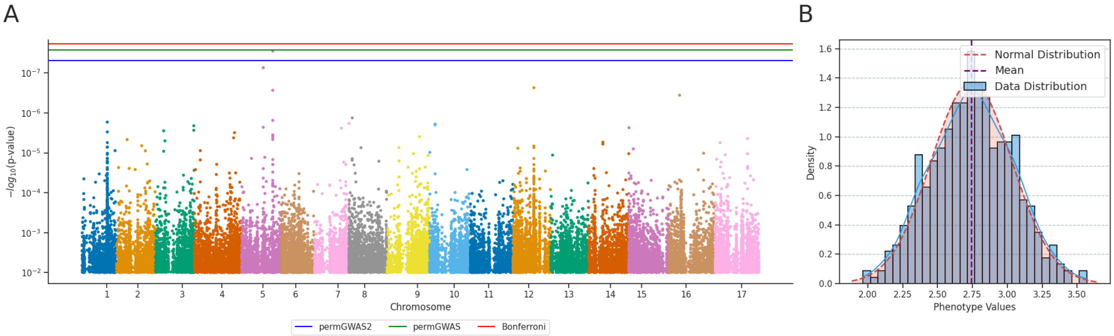


**Supplementary Figure 24: Manhattan and density plot of Seed maximum width[ANN rm218P103]:** (A) Manhattan plot with Bonferroni and two permutation-based thresholds. (B) Phenotypic distribution (Shapiro-Wilk p-value: 8.43975E-1)

**Supplementary Table 21: Significant Associations of Seed maximum width[ANN rm218P103]:** All significant associations found by at least one of the three thresholds permGWAS2 (perm2), permGWAS (perm), and Bonferroni (bonf). The columns show the chromosome, position and p-value of each hit. Additionally, the closest gene including the distance to the gene are shown. The last column contains the threshold(s) with respect to which the SNP was deemed significant.

| Chromosome | Position | P-value | Gene | Distance | Threshold |
|---|---|---|---|---|---|
| Ha412HOChr05 | 146526751 | 2.81955E-8 | Ha412HOChr05g0230551 | 0 | perm2 |

## Phenotype: Seed ovoid[ANN rm218P114]

| | | | |
|---|---|---|---|
| Number of samples: | 426 | `permGWAS2` Hits: | 1 |
| Number of SNPs: | 2682419 | `permGWAS` Hits: | 1 |
| Estimated heritability: | 7.10536E-1 | Bonferroni Hits: | 1 |

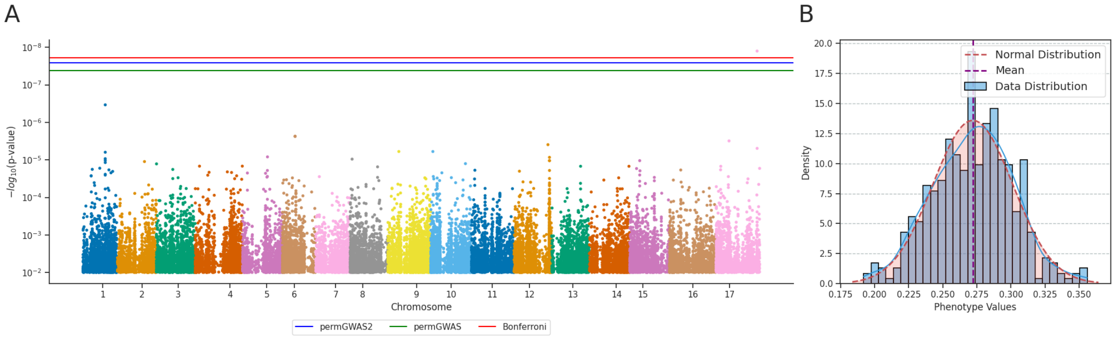


**Supplementary Figure 25: Manhattan and density plot of Seed ovoid[ANN rm218P114]:** (A) Manhattan plot with Bonferroni and two permutation-based thresholds. (B) Phenotypic distribution (Shapiro-Wilk p-value: 4.46124E-1)

**Supplementary Table 22: Significant Associations of Seed ovoid[ANN rm218P114]:** All significant associations found by at least one of the three thresholds `permGWAS2` (perm2), `permGWAS` (perm), and Bonferroni (bonf). The columns show the chromosome, position and p-value of each hit. Additionally, the closest gene including the distance to the gene are shown. The last column contains the threshold(s) with respect to which the SNP was deemed significant.

| Chromosome | Position | P-value | Gene | Distance | Threshold |
|---|---|---|---|---|---|
| Ha412HOChr17 | 192904025 | 1.25603E-8 | Ha412HOChr17g0853901 | 166627 | perm2,perm,bonf |

## Phenotype: Seed width mid height[ANN rm218P102]

| | | | |
|---|---|---|---|
| Number of samples: | 426 | permGWAS2 Hits: | 1 |
| Number of SNPs: | 2682419 | permGWAS Hits: | 1 |
| Estimated heritability: | 9.99955E-1 | Bonferroni Hits: | 0 |

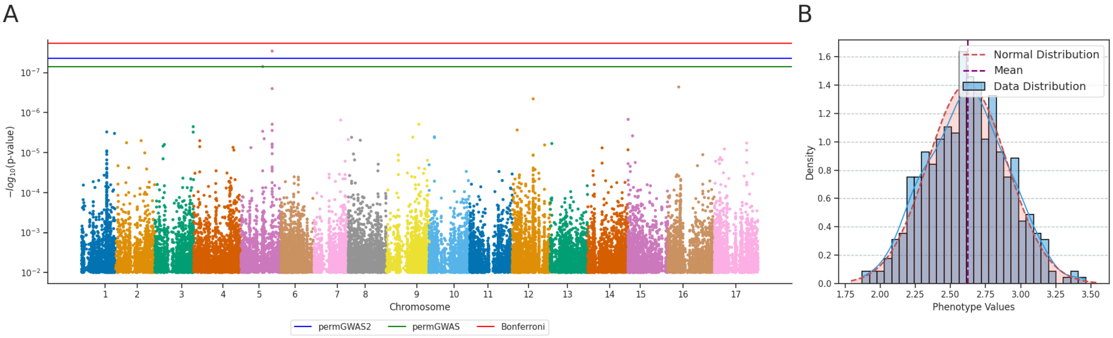


**Supplementary Figure 26: Manhattan and density plot of Seed width mid height[ANN rm218P102]:** (A) Manhattan plot with Bonferroni and two permutation-based thresholds. (B) Phenotypic distribution (Shapiro-Wilk p-value: 7.65497E-1)

**Supplementary Table 23: Significant Associations of Seed width mid height[ANN rm218P102]:** All significant associations found by at least one of the three thresholds permGWAS2 (perm2), permGWAS (perm), and Bonferroni (bonf). The columns show the chromosome, position and p-value of each hit. Additionally, the closest gene including the distance to the gene are shown. The last column contains the threshold(s) with respect to which the SNP was deemed significant.

| Chromosome | Position | P-value | Gene | Distance | Threshold |
|---|---|---|---|---|---|
| Ha412HOChr05 | 146526751 | 2.89665E-8 | Ha412HOChr05g0230551 | 0 | perm2,perm |

## Phenotype: Stem colour[ANN rm218P133]

| | | | |
|---|---|---|---|
| Number of samples: | 614 | `permGWAS2` Hits: | 1 |
| Number of SNPs: | 2677290 | `permGWAS` Hits: | 1 |
| Estimated heritability: | 8.91461E-1 | Bonferroni Hits: | 0 |

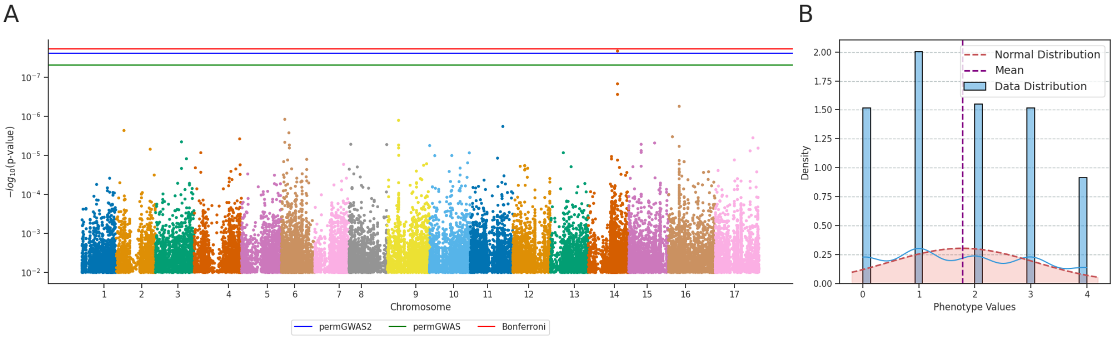


**Supplementary Figure 27: Manhattan and density plot of Stem colour[ANN rm218P133]:** (A) Manhattan plot with Bonferroni and two permutation-based thresholds. (B) Phenotypic distribution (Shapiro-Wilk p-value: 9.73083E-20)

**Supplementary Table 24: Significant Associations of Stem colour[ANN rm218P133]:** All significant associations found by at least one of the three thresholds `permGWAS2` (perm2), `permGWAS` (perm), and Bonferroni (bonf). The columns show the chromosome, position and p-value of each hit. Additionally, the closest gene including the distance to the gene are shown. The last column contains the threshold(s) with respect to which the SNP was deemed significant.

| Chromosome | Position | P-value | Gene | Distance | Threshold |
|---|---|---|---|---|---|
| Ha412HOChr14 | 137463531 | 2.14628E-8 | Ha412HOChr14g0668411 | 45409 | perm2,perm |

## Phenotype: TLN

| | | | |
|---|---|---|---|
| Number of samples: | 605 | permGWAS2 Hits: | 106 |
| Number of SNPs: | 2679385 | permGWAS Hits: | 32 |
| Estimated heritability: | 9.99955E-1 | Bonferroni Hits: | 72 |

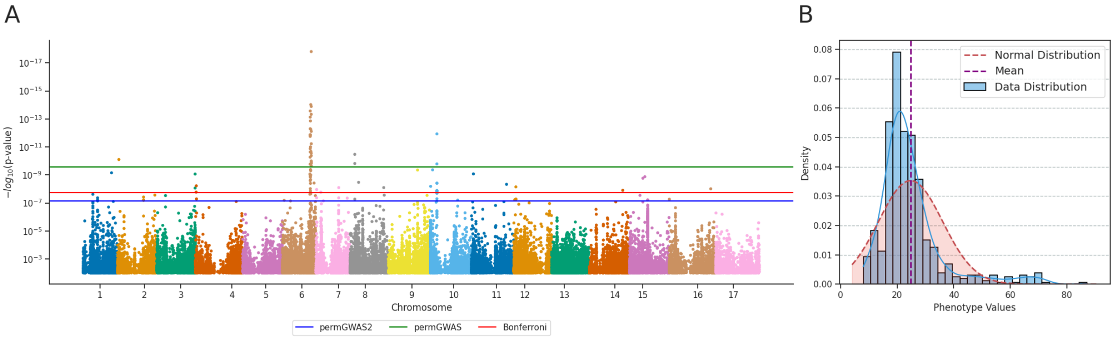


**Supplementary Figure 28: Manhattan and density plot of TLN:** (A) Manhattan plot with Bonferroni and two permutation-based thresholds. (B) Phenotypic distribution (Shapiro-Wilk p-value: 6.22241E-28)

**Supplementary Table 25: Significant Associations of TLN:** Significant associations within or close to a gene (distance $\leq$ 200 bp) found by at least one of the three thresholds permGWAS2 (perm2), permGWAS (perm), and Bonferroni (bonf). The columns show the chromosome, position and p-value of each hit. Additionally, the closest gene including the distance to the gene are shown. The last column contains the threshold(s) with respect to which the SNP was deemed significant.

| Chromosome | Position | P-value | Gene | Distance | Threshold |
|---|---|---|---|---|---|
| Ha412HOChr01 | 45110583 | 2.39974E-8 | Ha412HOChr01g0010021 | 0 | perm2 |
| Ha412HOChr01 | 132685321 | 7.06228E-10 | Ha412HOChr01g0034671 | 0 | perm2,bonf |
| Ha412HOChr02 | 123945736 | 5.99910E-8 | Ha412HOChr02g0073361 | 0 | perm2 |
| Ha412HOChr02 | 176986053 | 2.72675E-8 | Ha412HOChr02g0087391 | 0 | perm2 |
| Ha412HOChr03 | 42467534 | 2.97273E-8 | Ha412HOChr03g0102261 | 0 | perm2 |
| Ha412HOChr03 | 181204493 | 8.62760E-9 | Ha412HOChr03g0144601 | 25 | perm2,bonf |
| Ha412HOChr03 | 181293779 | 8.61355E-10 | Ha412HOChr03g0144781 | 135 | perm2,bonf |
| Ha412HOChr04 | 4467336 | 1.67571E-8 | Ha412HOChr04g0147001 | 0 | perm2,bonf |
| Ha412HOChr04 | 5208771 | 5.95623E-9 | Ha412HOChr04g0147311 | 0 | perm2,bonf |
| Ha412HOChr06 | 130730537 | 2.70920E-14 | Ha412HOChr06g0281381 | 0 | perm2,perm,bonf |
| Ha412HOChr07 | 4550495 | 4.22238E-8 | Ha412HOChr07g0288531 | 0 | perm2 |
| Ha412HOChr07 | 4550778 | 1.09099E-8 | Ha412HOChr07g0288531 | 0 | perm2,bonf |
| Ha412HOChr07 | 24292548 | 1.68711E-8 | Ha412HOChr07g0292821 | 0 | perm2,bonf |
| Ha412HOChr07 | 147858991 | 4.50575E-8 | Ha412HOChr07g0318701 | 0 | perm2 |
| Ha412HOChr08 | 21801953 | 5.30677E-8 | Ha412HOChr08g0337821 | 0 | perm2 |
| Ha412HOChr08 | 157493582 | 7.97387E-9 | Ha412HOChr08g0368891 | 0 | perm2,bonf |
| Ha412HOChr08 | 159909069 | 2.75862E-8 | Ha412HOChr08g0369371 | 0 | perm2 |
| Ha412HOChr09 | 183780555 | 2.86348E-8 | Ha412HOChr09g0420451 | 0 | perm2 |
| Ha412HOChr10 | 9666517 | 4.30153E-10 | Ha412HOChr10g0433571 | 0 | perm2,bonf |
| Ha412HOChr12 | 10328784 | 7.09344E-9 | Ha412HOChr12g0539451 | 0 | perm2,bonf |
| Ha412HOChr12 | 10354219 | 5.39585E-8 | Ha412HOChr12g0539461 | 167 | perm2 |
| Ha412HOChr12 | 10354847 | 5.20196E-8 | Ha412HOChr12g0539461 | 0 | perm2 |

| | | | | | |
|---|---|---|---|---|---|
| Ha412HOChr15 | 49160405 | 2.84829E-8 | Ha412HOChr15g0713071 | 0 | perm2 |
| Ha412HOChr16 | 1298169 | 5.37259E-8 | Ha412HOChr16g0747151 | 0 | perm2 |
| Ha412HOChr16 | 195294782 | 9.69983E-9 | Ha412HOChr16g0795651 | 0 | perm2,bonf |

## Phenotype: Trichomes density edge average[ANN rm218P181]

| | | | |
|---|---|---|---|
| Number of samples: | 596 | `permGWAS2` Hits: | 0 |
| Number of SNPs: | 2680680 | `permGWAS` Hits: | 0 |
| Estimated heritability: | 1.41355E-1 | Bonferroni Hits: | 1 |

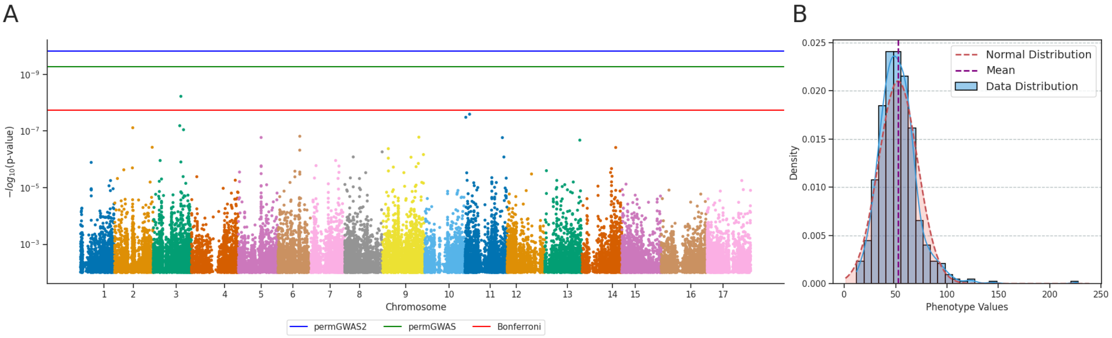


**Supplementary Figure 29: Manhattan and density plot of Trichomes density edge average[ANN rm218P181]:** (A) Manhattan plot with Bonferroni and two permutation-based thresholds. (B) Phenotypic distribution (Shapiro-Wilk p-value: 1.94740E-19)

**Supplementary Table 26: Significant Associations of Trichomes density edge average[ANN rm218P181]:** All significant associations found by at least one of the three thresholds `permGWAS2` (perm2), `permGWAS` (perm), and Bonferroni (bonf). The columns show the chromosome, position and p-value of each hit. Additionally, the closest gene including the distance to the gene are shown. The last column contains the threshold(s) with respect to which the SNP was deemed significant.

| Chromosome | Position | P-value | Gene | Distance | Threshold |
|---|---|---|---|---|---|
| Ha412HOChr03 | 131245450 | 5.93487E-9 | Ha412HOChr03g0121921 | 3619 | bonf |

## Phenotype: Trichomes density leaf center secondary veins[ANN rm218P180]

| | | | |
|---|---|---|---|
| Number of samples: | 596 | permGWAS2 Hits: | 0 |
| Number of SNPs: | 2680680 | permGWAS Hits: | 0 |
| Estimated heritability: | 2.99819E-1 | Bonferroni Hits: | 2 |

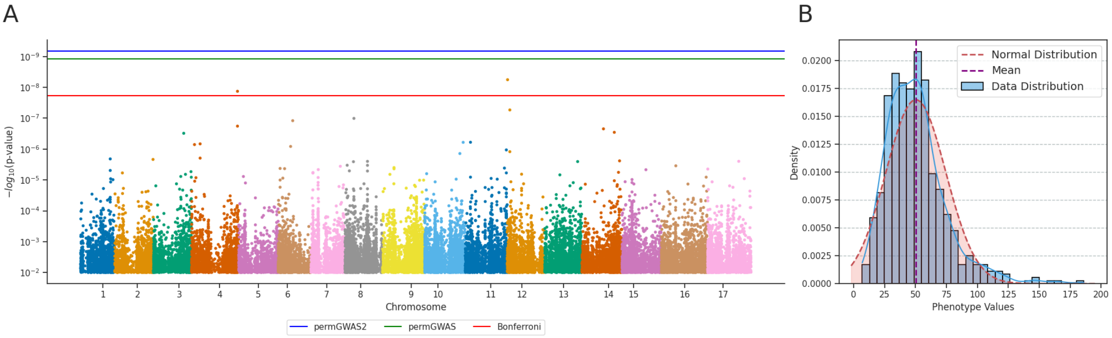


**Supplementary Figure 30: Manhattan and density plot of Trichomes density leaf center secondary veins[ANN rm218P180]:** (A) Manhattan plot with Bonferroni and two permutation-based thresholds. (B) Phenotypic distribution (Shapiro-Wilk p-value: 5.51221E-18)

**Supplementary Table 27: Significant Associations of Trichomes density leaf center secondary veins[ANN rm218P180]:** All significant associations found by at least one of the three thresholds permGWAS2 (perm2), permGWAS (perm), and Bonferroni (bonf). The columns show the chromosome, position and p-value of each hit. Additionally, the closest gene including the distance to the gene are shown. The last column contains the threshold(s) with respect to which the SNP was deemed significant.

| Chromosome | Position | P-value | Gene | Distance | Threshold |
|---|---|---|---|---|---|
| Ha412HOChr04 | 216216819 | 1.34414E-8 | Ha412HOChr04g0196381 | 3866 | bonf |
| Ha412HOChr12 | 1621071 | 5.66482E-9 | Ha412HOChr12g0534351 | 4342 | bonf |

## Phenotype: Trichomes density leaf edge flat area[ANN rm218P177]

| | | | |
|---|---|---|---|
| Number of samples: | 596 | `permGWAS2` Hits: | 0 |
| Number of SNPs: | 2680680 | `permGWAS` Hits: | 0 |
| Estimated heritability: | 1.60520E-1 | Bonferroni Hits: | 1 |

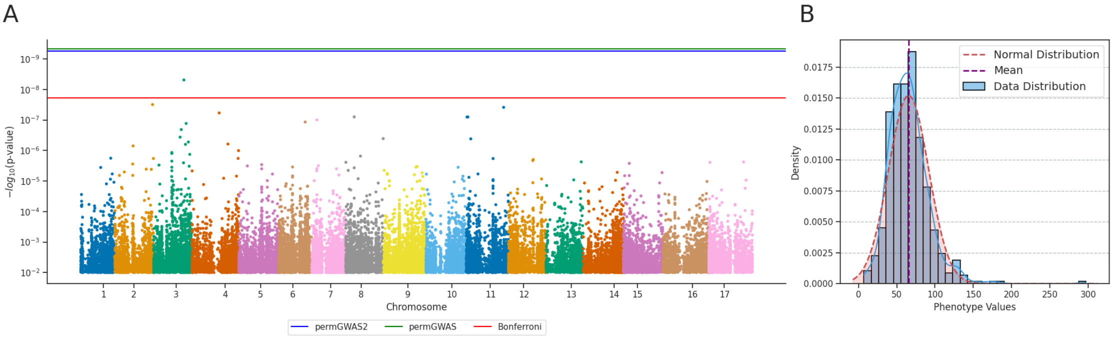

**Supplementary Figure 31: Manhattan and density plot of Trichomes density leaf edge flat area[ANN rm218P177]:** (A) Manhattan plot with Bonferroni and two permutation-based thresholds. (B) Phenotypic distribution (Shapiro-Wilk p-value: 1.94122E-18)

**Supplementary Table 28: Significant Associations of Trichomes density leaf edge flat area[ANN rm218P177]:** All significant associations found by at least one of the three thresholds `permGWAS2` (perm2), `permGWAS` (perm), and Bonferroni (bonf). The columns show the chromosome, position and p-value of each hit. Additionally, the closest gene including the distance to the gene are shown. The last column contains the threshold(s) with respect to which the SNP was deemed significant.

| Chromosome | Position | P-value | Gene | Distance | Threshold |
|---|---|---|---|---|---|
| Ha412HOChr03 | 143795931 | 4.87764E-9 | Ha412HOChr03g0126421 | 63417 | bonf |

### Phenotype: Trichomes density leaf edge secondary veins[ANN rm218P178]

| | | | |
|---|---|---|---|
| Number of samples: | 596 | permGWAS2 Hits: | 0 |
| Number of SNPs: | 2680680 | permGWAS Hits: | 0 |
| Estimated heritability: | 1.95402E-1 | Bonferroni Hits: | 7 |

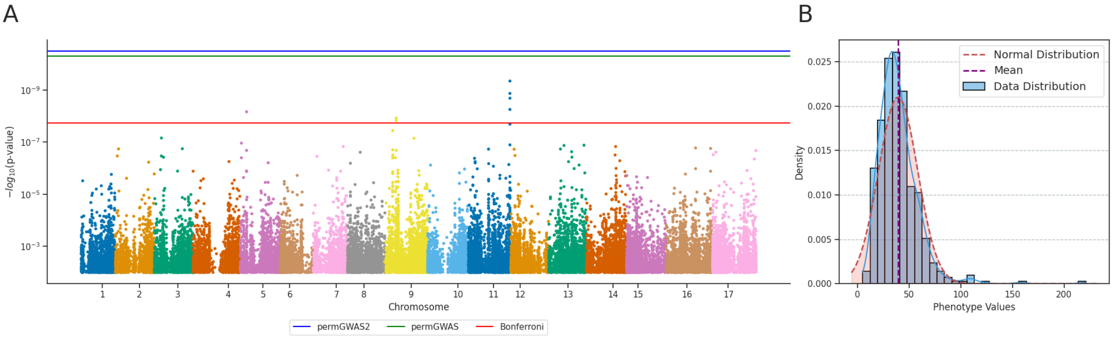


**Supplementary Figure 32: Manhattan and density plot of Trichomes density leaf edge secondary veins[ANN rm218P178]:** (A) Manhattan plot with Bonferroni and two permutation-based thresholds. (B) Phenotypic distribution (Shapiro-Wilk p-value: 7.74256E-24)

**Supplementary Table 29: Significant Associations of Trichomes density leaf edge secondary veins[ANN rm218P178]:** All significant associations found by at least one of the three thresholds permGWAS2 (perm2), permGWAS (perm), and Bonferroni (bonf). The columns show the chromosome, position and p-value of each hit. Additionally, the closest gene including the distance to the gene are shown. The last column contains the threshold(s) with respect to which the SNP was deemed significant.

| Chromosome | Position | P-value | Gene | Distance | Threshold |
|---|---|---|---|---|---|
| Ha412HOChr05 | 29970141 | 6.87181E-9 | Ha412HOChr05g0208891 | 0 | bonf |
| Ha412HOChr09 | 52031318 | 1.48478E-8 | Ha412HOChr09g0383801 | 86670 | bonf |
| Ha412HOChr09 | 52087741 | 1.20808E-8 | Ha412HOChr09g0383841 | 64614 | bonf |
| Ha412HOChr11 | 194869636 | 5.58674E-9 | Ha412HOChr11g0531281 | 24187 | bonf |
| Ha412HOChr11 | 194869648 | 1.35859E-9 | Ha412HOChr11g0531281 | 24175 | bonf |
| Ha412HOChr11 | 194869653 | 2.06634E-9 | Ha412HOChr11g0531281 | 24170 | bonf |
| Ha412HOChr11 | 194869668 | 4.54801E-10 | Ha412HOChr11g0531281 | 24155 | bonf |

## Phenotype: Trichomes density vein average[ANN rm218P184]

| | | | |
|---|---|---|---|
| Number of samples: | 596 | `permGWAS2` Hits: | 0 |
| Number of SNPs: | 2680680 | `permGWAS` Hits: | 0 |
| Estimated heritability: | 3.11496E-1 | Bonferroni Hits: | 1 |

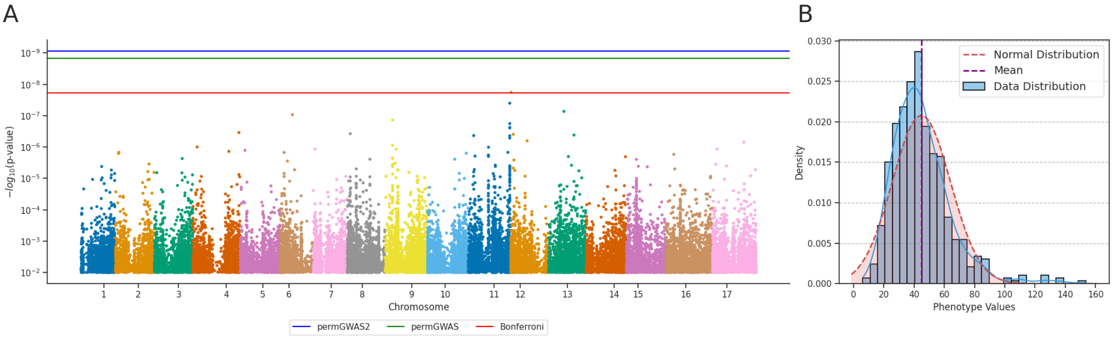


**Supplementary Figure 33: Manhattan and density plot of Trichomes density vein average[ANN rm218P184]:** (A) Manhattan plot with Bonferroni and two permutation-based thresholds. (B) Phenotypic distribution (Shapiro-Wilk p-value: 1.36663E-18)

**Supplementary Table 30: Significant Associations of Trichomes density vein average[ANN rm218P184]:** All significant associations found by at least one of the three thresholds `permGWAS2` (perm2), `permGWAS` (perm), and Bonferroni (bonf). The columns show the chromosome, position and p-value of each hit. Additionally, the closest gene including the distance to the gene are shown. The last column contains the threshold(s) with respect to which the SNP was deemed significant.

| Chromosome | Position | P-value | Gene | Distance | Threshold |
|---|---|---|---|---|---|
| Ha412HOChr12 | 1621071 | 1.78962E-8 | Ha412HOChr12g0534351 | 4342 | bonf |
